# A radiomics nomogram prediction for survival of patients with “driver gene-negative” lung adenocarcinomas (LUAD)

**DOI:** 10.1007/s11547-023-01643-4

**Published:** 2023-05-23

**Authors:** Qi-Kun Guo, Hao-Shuai Yang, Shi-Chao Shan, Dan-Dan Chang, Li-Jie Qiu, Hong-He Luo, He-Ping Li, Zun-Fu Ke, Ying Zhu

**Affiliations:** 1 Department of Oncology, The Affiliated He Xian Memorial Hospital of Southern Medical University, Guangzhou, 510080 Province Guangdong People’s Republic of China; 2 Department of Interventional Radiology, The First Affiliated Hospital of Sun Yat-Sen University, Guangzhou, 510080 Province Guangdong People’s Republic of China; 3 Department of Thoracic Surgery, China-Japan Friendship Hospital, Beijing, China; 4 Department of Thoracic Surgery, The First Affiliated Hospital, Sun Yat-Sen University, Guangzhou, 510080 Province Guangdong People’s Republic of China; 5 Department of Radiology, The First Affiliated Hospital, Sun Yat-Sen University, Guangzhou, 510080 Province Guangdong People’s Republic of China; 6 Department of Medical Oncology of the Eastern Hospital, The First Affiliated Hospital of Sun Yat-Sen University, Guangzhou, 510080 People’s Republic of China; 7 Department of Pathology, The First Affiliated Hospital, Sun Yat-Sen University, Guangzhou, 510080 Province Guangdong People’s Republic of China; 8 Institution of Precision Medicine, The First Affiliated Hospital, Sun Yat-Sen University, Guangzhou, 510080 Province Guangdong People’s Republic of China

**Keywords:** Driver gene-negative, Lung adenocarcinoma, X-ray computed tomography, Radiomics, Biological pathways

## Abstract

**Background:**

To study the role of computed tomography (CT)-derived radiomics features and clinical characteristics on the prognosis of “driver gene-negative” lung adenocarcinoma (LUAD) and to explore the potential molecular biological which may be helpful for patients’ individual postoperative care.

**Methods:**

A total of 180 patients with stage I-III “driver gene-negative” LUAD in the First Affiliated Hospital of Sun Yat-Sen University from September 2003 to June 2015 were retrospectively collected. The Least Absolute Shrinkage and Selection Operator (LASSO) Cox regression model was used to screen radiomics features and calculated the Rad-score. The prediction performance of the nomogram model based on radiomics features and clinical characteristics was validated and then assessed with respect to calibration. Gene set enrichment analysis (GSEA) was used to explore the relevant biological pathways.

**Results:**

The radiomics and the clinicopathological characteristics were combined to construct a nomogram resulted in better performance for the estimation of OS (C-index: 0.815; 95% confidence interval [CI]: 0.756–0.874) than the clinicopathological nomogram (C-index: 0.765; 95% CI: 0.692–0.837). Decision curve analysis demonstrated that in terms of clinical usefulness, the radiomics nomogram outperformed the traditional staging system and the clinicopathological nomogram. The clinical prognostic risk score of each patient was calculated based on the radiomics nomogram and divided by X-tile into high-risk (> 65.28) and low-risk (≤ 65.28) groups. GSEA results showed that the low-risk score group was directly related to amino acid metabolism, and the high-risk score group was related to immune and metabolism pathways.

**Conclusions:**

The radiomics nomogram was promising to predict the prognosis of patients with “driver gene-negative” LUAD. The metabolism and immune-related pathways may provide new treatment orientation for this genetically unique subset of patients, which may serve as a potential tool to guide individual postoperative care for those patients.

**Supplementary Information:**

The online version contains supplementary material available at 10.1007/s11547-023-01643-4.

## Introduction

Lung cancer is the second most common malignant tumor but the most common cause of malignancy-related mortality worldwide [1]. More than 80% of histological types of lung cancer are lung adenocarcinoma (LUAD) [2]. Over the last decade, targeted therapy has greatly prolonged the overall survival of driver gene-mutated lung cancer patients [3, 4]. In addition to EGFR mutations and ALK fusions, driver genes also include KRAS, BRAF and HER2, as well as gene rearrangements involving RET, ROS1 and MET exon 14 skipping [5, 6]. However, 14–25% of LUAD patients are still “driver gene-negative” [5], which identified as patients with LUAD negative for EGFR, KRAS, BRAF, HER2, MET, ALK, RET and ROS1 [3]. And with the development of large-panel sequencing technology, this proportion is increasing. Although immune checkpoint inhibitors (ICIs) such as anti-programmed death-1 (PD-1)/PD-ligand-1 (PD-L1) have shown great potential in the treatment of lung cancer [7], the large number of non-responders and the immune-related toxicities of ICIs limits the application of immunotherapy in LUAD [8]. From the current research results, the first-line pembrolizumab monotherapy demonstrated an OS benefit than chemotherapy for patients with advanced non-small cell lung cancer who have not been treated before and without EGFR/ALK aberrations [9], whereas, for patients with “driving gene-negative” LUAD, the treatment option is limited [5] and the efficacy of ICIs still needs further exploration [9, 10].

Medical imaging technology plays an important role in clinical management. By extracting multiple quantitative features from conventional computed tomography (CT) images, radiomics could capture the differences between different tumor phenotypes non-invasively [11]. Recent advances in radiomics showed that it has provided potential usefulness personalized insights increasingly in oncologic practice such as tumor detection, subtype classification, prognosis and treatment response assessment [12–14]. The nomogram obtained by combining radiomics with clinicopathological characteristics appears to improve the accuracy of prognosis prediction [15]. However, the radiomic nomogram associated with the prognosis of “driver gene-negative” LUAD has not yet been described.

Therefore, the purpose of the present study was to build a nomogram based on radiomics CT features and clinical characteristics to predict the survival of patients with “driver gene-negative” LUAD and to evaluate the incremental value of radiomic signatures to traditional staging systems and clinicopathological risk factors. In addition, we intended to use radiomics nomogram and gene expression data to explore the potential molecular biological in order to guide individual postoperative care for those patients.

## Materials and methods

### Patients

This study was approved by the Ethics Committee of The First Affiliated Hospital of Sun Yat-Sen University with a waiver of informed consent (No. [2021]531; date of approval: 20/08/2021). The data of bulk RNA-seq had been uploaded to the Gene Expression Omnibus (GEO) database by our previous published article [3]. We selected 371 “driver gene-negative” LUAD patients in the First Affiliated Hospital of Sun Yat-Sen University from September 2003 to June 2015. None of the patients underwent any antitumor therapy before biopsy sampling, and "driver gene–negative" status was determined in paraffin-embedded (FFPE) tissues. A total of 60 pairs of fresh tumor and adjacent normal tissues were selected randomly from the 371 patients with “driver gene–negative” LUAD and were used for genome-wide microarray assay to screen candidate genes followed by Western blotting and qPCR. Circulating tumor cell (CTC) enumeration and PD-L1 expression detection were performed on each eligible patient. A total of 191 patients were excluded from this study because without surgical resection and poor CT image quality.

The final patient cohort was 180 patients from June 2007 to June 2015 after screening by strict inclusion and exclusion criteria. Only 28 patients’ fresh tumors and adjacent normal tissues sequencing results were available, including 11 patients of stage I, 5 patients of stage II and 12 patients of stage III. All chest CT images with an 1-mm axial reconstruction interval were acquired within 2 weeks (median value of 6 days). And then, the training and validation data sets were assigned 7-to-3, and the latter included the 28 patients with available genetic data. The workflow is shown in Supplementary Fig. 1.

The endpoint of this study was OS, which was defined as the time from the date of pathological diagnosis to death or the last follow-up. All patients were followed up for at least five years, unless the patient died. Baseline clinical–pathological data, such as age, sex, smoking status, stage, histologic grade, were obtained from the medical records (Table 1).

**Table 1:** Characteristics of Patients with “driver gene-negative” LUAD in the Training Dataset and Validation Dataset

Characteristic	Training data set (*n* = 129)	Validation data set (*n* = 51)	*p* value
Gender			0.713
Male	72(55.8)	30(58.8)	
Female	57(44.2)	21(41.2)	
Age			0.855
Mean ± SD(years)	58.62 ± 7.89	57.82 ± 7.42	
Range	35 ~ 81	38 ~ 76	
≤ 60	99(76.8)	41(80.4)	
61–69	19(14.7)	6(11.8)	
≥ 70	11(8.5)	4(7.8)	
Smoking status			0.984
No	101(78.3)	40(78.4)	
Yes	28(21.7)	11(21.6)	
Stage			0.070
I	69(53.5)	27(52.9)	
II	39(30.2)	9(17.7)	
III	21(16.3)	15(29.4)	
T stage			0.105
1	46(35.7)	17(33.3)	
2	81(62.8)	30(58.8)	
3	2(1.5)	2(3.9)	
4	0(0)	2(3.9)	
N stage			0.112
0	103(79.8)	38(74.5)	
1	11(8.5)	2(3.9)	
2	13(10.1)	7(13.7)	
3	2(1.6)	4(7.8)	
Degrees of differentiation			0.800
High	8(6.2)	2(3.9)	
Moderate	101(78.3)	40(78.5)	
Low	20(15.5)	9(17.6)	
CTC count			0.251
Mean ± SD	3.85 ± 4.56	2.90 ± 3.79	
Range	0 ~ 20	0 ~ 12	
< 4	74(57.4)	34(66.6)	
≥ 4	55(42.6)	17(33.3)	
PD-L1 expression			0.544
Low	101(78.3)	42(82.3)	
High	28(21.7)	9(17.7)	
Rad-score			0.735
Mean ± SD	0.02 ± 0.46	− 0.03 ± 0.36	
Range	-0.72 ~ 2.14	− 0.79 ~ 1.22	
Overall survival(month)			0.801
Mean ± SD	50.95 ± 22.40	52.49 ± 29.87	
Range	9 ~ 120	18 ~ 162	

*LUAD* lung adenocarcinoma, *CTC* circulating tumor cell, *SD* standard deviation, *PD-L1* programmed death ligand-1, *Rad-score* radiomics score

### PD-L1 protein expression detection and CTC enumeration

Immunohistochemical analysis of PD-L1 expression was detected with primary rabbit monoclonal antibody against human PD-L1 (SP263; 1:2000; Roche Ventana, Tucson, AZ, USA) in deparaffinized and hydrated tumor tissue. PD-L1 expression depended on the intensity of cell membrane staining. The proportion of PD-L1-positive cells was independently estimated as the percentage of total tumor cells in whole sections by two pathologists. If the independent assessments did not agree, the slides were reviewed by the two investigators together to achieve consensus. The consensus judgments were adopted as the final results. The PD-L1 expression is defined by tumor proportion scores (TPS). PD-L1 TPS ≥ 1% was defined as positive expression, and PD-L1 TPS ≥ 50% was defined as high expression.

The methodology of CTC enumeration was detailed in our previous study [16].

### Image acquisition and radiomics processing

As for the acquisition parameters and retrieval procedures of CT images in our study, more details are presented in Supplementary material E1. Tumors were delineated by the junior radiologist (with 6 years of clinical experience in chest CT study interpretation) on the CT images using the same active-contour semiautomatic algorithm and verified by the expert radiologists (with 12 years of clinical experience in chest CT study interpretation) through the ITK-SNAP software (version 3.8.0, https://www.itksnap.org). Radiomics features were extracted from CT images resampled to isometric voxels of 1 × 1 × 1 mm^3^ by using the PyRadiomics platform [17] implemented in Python software (version 3.8.3, https://www.python.org). The details of the platform and radiomics features are described in Supplementary material E2. Totally 1409 radiomics features were, respectively, extracted from plain and enhanced CT images, including 14 shape features, 18 first-order intensity statistics features and 75 texture features (Table S1), as well as 558 Laplacian of Gaussian (LOG) features, 744 wavelet features.

### Radiomics features selection and radiomics score calculation

A two-way random, single-measure (absolute agreement) intraclass correlation coefficient (ICC) was used to evaluate the robustness of the extracted radiomics features. If the ICC value was greater than 0.8 in the training data set, then the stability of the feature can be considered excellent [18].

The variables included in the multivariate analysis should follow Harrell's guidelines [19], that is, the number of events of interest should exceed the number of variables by at least 5 times. However, including more variables does not necessarily lead to higher accuracy, but leads to overfitting [20]. To further remove redundant features in the training data set, the Least Absolute Shrinkage and Selection Operator (LASSO) Cox regression model [21, 22], a popular regularized machine learning algorithm suitable for dimensionality reduction of high-dimensional data, was used to select the most useful radiomics characteristics which related to prognosis with tenfold nested cross-validation. The selected radiomics features were then weighted by their respective coefficients and summed to get the radiomics score (Rad-score) of each patient [23].

### Radiomics nomogram construction

The clinical pathological data of the training data set were analyzed by single-factor Cox regression analysis to evaluate the potential relationship between each selected feature and OS. And then, the independent clinical–pathological risk factors were selected by the multivariate Cox analysis and used to construct a clinicopathological nomogram.

Integrated the selected independent clinicopathological risk factors and the Rad-score into the Cox regression model, and construct a radiomics nomogram via the training set. In order to quantify the prognostic risk, the clinical prognostic score of patients in the validation data set was calculated by the nomogram. Calibration curves were used to compare the consistency between the observed results and the OS correlation of clinicopathological nomogram and radiomics nomogram.

As for the evaluation of gain effect whether the radiomics characteristics would have on the clinical prognosis prediction performance of the clinical pathological factor model, the Harrell Concordance Index (C-index) was calculated. The improvement of usefulness brought by the radiomics feature was quantitatively calculated using the integrated discrimination improvement (IDI) [24], which is very sensitive in detecting prediction probability changes in a new model compared to an old model. The Akaike information criteria (AIC) were used to assess the risk of overfitting [25]. And then, calculate the net income under different threshold probabilities and draw a decision curve.

X-tile [26] (version 3.6.1, Yale University School of Medicine, New Haven, Conn) was used to determine the cutoff value of the prognostic score, and patients were divided into high-risk groups and low-risk groups. The difference in survival curves between the high-risk group and the low-risk group was evaluated by a weighted log-rank test (G-rho rank test, rho = 1) [27]. Time-dependent areas under the receiver operating characteristic curve (AUROC) for OS could also be generated by using the multivariable model.

### Genetic studies of the radiomics nomogram

In order to explore the biological basis of the prognostic risk score grouping obtained by the radiomics nomogram, it was necessary to conduct related evaluations of potential molecular biology pathways. We used the 4 × 44 K whole human genome expression microarray (Agilent design ID 026,652, GEO accession number GPL13497) to get profiles the expression of 27,958 genes among the 28 pairs of qualified samples. We used GeneSpring GX v12.1 software package to do quantile normalization and subsequent data processing, and gene with low expression or close to the background level was excluded for analyses.

We preformed pre-ranked gene set enrichment analysis (GSEA) approach for genetic analysis as in previously published studies [28–30]. Gene set enrichment analysis (GSEA) derives its power by focusing on gene sets, that is, groups of genes that share common biological function, chromosomal location, or regulation [31]. Gene expression values were correlated with the prognostic risk score to rank all genes by using Spearman’s rank correlation coefficient, and we put this gene rank into a ranked gene set enrichment analysis (GSEA) software version 4.2.0. We tested expert-curated pathways from C2 Reactome collection available at MSigDB [32] database cp.kegg.v72.symbols GMT data set. The weighted enrichment analysis method in GSEA was used to conduct enrichment analysis by random combination of 1000 times, and adj. *p* value was calculated as per to correct for multiple hypothesis testing. The normalized enrichment score (NES) of GSEA software was used to quantify the correlation of the genes with pathways.

**Fig. 1:**
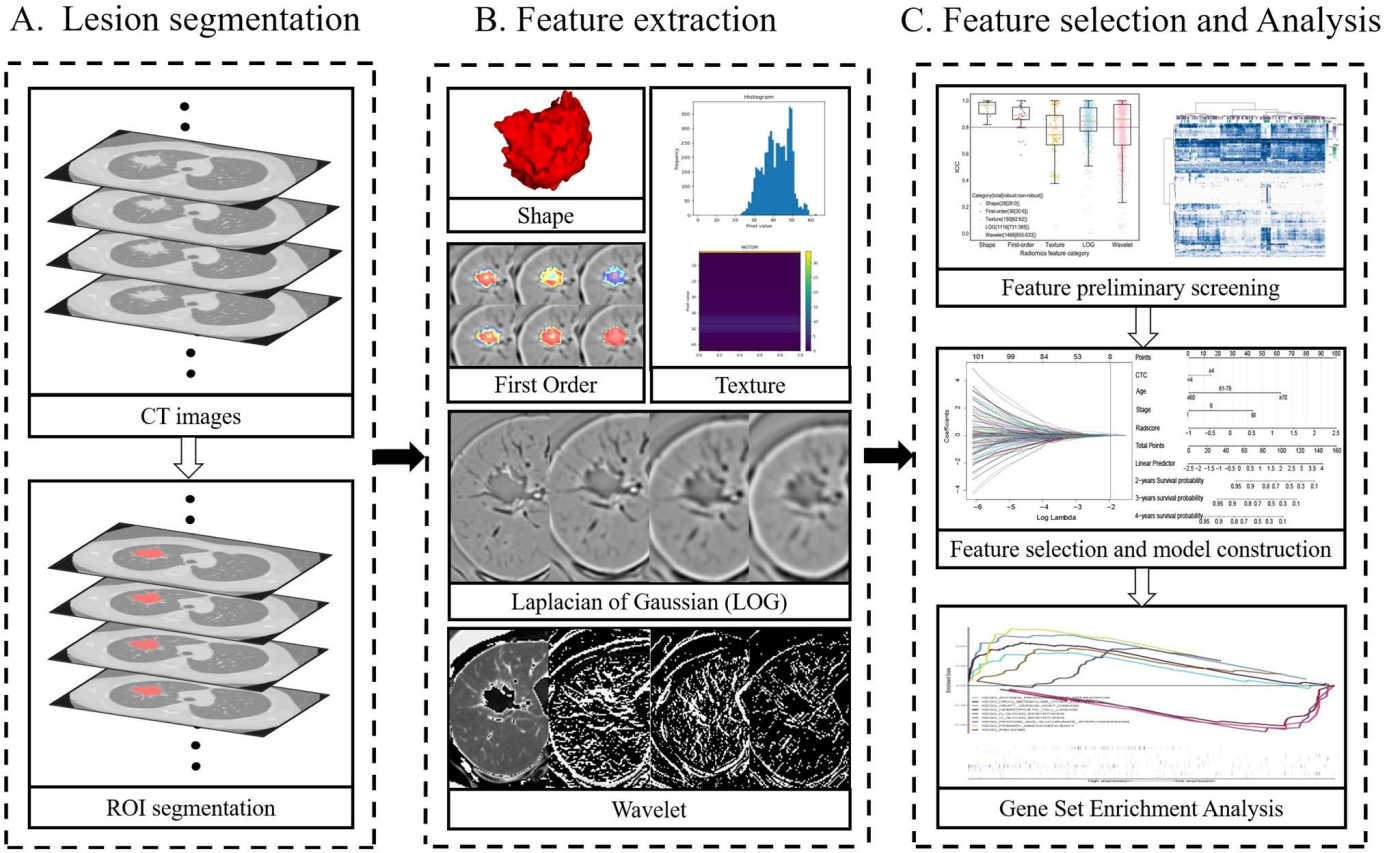
Flow chart of radiomics. **A**. Acquisition image data and segment the region of interest for the entire tumor; **B**. extract multiple quantitative features from the CT image, quantify the shape, first order, texture, Laplacian of Gaussian (LOG) texture and wavelet texture of the tumor-related information. **D**. After multiple screening, radiomics characteristics were comprehensively analyzed with clinical data and gene expression data

The overview of our pipeline is given in Fig. 1.

### Statistical analysis

The statistical analysis was performed with R software (version 4.0.4, https://www.R-project.org). The packages in R used in this study are described in Supplementary material E3.

In order to evaluate the difference of the clinical information between the training and validation data sets, the Mann–Whitney U test was used for continuous variables (mean survival time) and the classification variables (gender, age, stage, smoking status, degrees of differentiation, CTC count, PD-L1 expression) using *χ*2 test (Table 1). The levels of statistical significance reported were double-sided, and *p* value < 0.05 was considered as significant.

## Results

### Patient clinical characteristics

The clinical characteristics of the training data set and the validation data set are shown in Table 1. A total of 180 patients (102 male and 78 female patients; mean age, 58.39 years ± 7.75; range, 35–81 years) were included in this study (Table 1). The difference was not statistically significant in terms of clinical characteristics including gender, age, smoking status, stage, tumor differentiation, CTC count, PD-L1 expression, Rad-score and OS.

Univariate Cox Proportional Hazards Regression Analysis (Table 2) in the training data set showed that the differences of age, stage, CTC count and Rad-score were statistically significant with *p* < 0.001.

**Table 2:** Univariate Cox Proportional Hazards Regression Analysis in Patients with “driver gene-negative” LUAD

Characteristic	Univariate analysis		
	Hazard ratio	95% CI	*p* value
Gender			
Male	Reference		
Female	1.06	0.94–0.65	0.81
Age	1.04	1.02–1.06	< 0.001*
≤ 60	Reference		
61–69	3.12	1.67–5.79	< 0.001*
≥ 70	6.36	3.05–13.27	< 0.001*
Smoking status			
No	Reference		
Yes	1.17	0.65–2.11	0.59
Stage			
I	Reference		
II	1.10	0.62–1.94	0.75
III	4.31	2.21–8.40	< 0.001*
Degrees of differentiation			
High	Reference		
Moderate	1.15	0.35–3.71	0.82
Low	1.19	0.32–4.33	0.79
CTC count			
< 4	Reference		
⩾4	2.62	1.59–4.33	< 0.001*
PD-L1 expression			
Low	Reference		
High	1.52	0.86–2.72	0.15
Rad-score	3.18	1.97–5.15	< 0.001*

*LUAD* lung adenocarcinoma, *CI* confidence interval, *CTC* circulating tumor cell, *SD* standard deviation, *PD-L1* programmed death ligand-1, *Rad-score* radiomics score

^*^statistically significant *p* value

### Radiomics features selection and radiomics score calculation

Selecting all features with high stability (ICC > 0.8) among 180 patients resulted in 1706 radiomics features (28 shape features, 30 first-order intensity statistics features, 62 texture features, 731 LoG features and 855 wavelet features) (Supplementary Fig. 2). And then, the retained features were further screened using the LASSO-Cox method (Supplementary Fig. 3). Finally, 7 radiomics features with nonzero weighting coefficients were obtained (Supplementary Fig. 4), and the corresponding rad-scores were calculated, respectively, (Supplementary Fig. 5). The calculation formula is shown in Supplementary material E4.

There was no statistical difference between the rad-scores in the training set and the validation set (*p* = 0.735) (Table 1), and it was an independent risk factor for prognosis (hazard ratio (HR) = 3.18) (Table 2).

### Radiomics nomogram construction

**Fig. 2:**
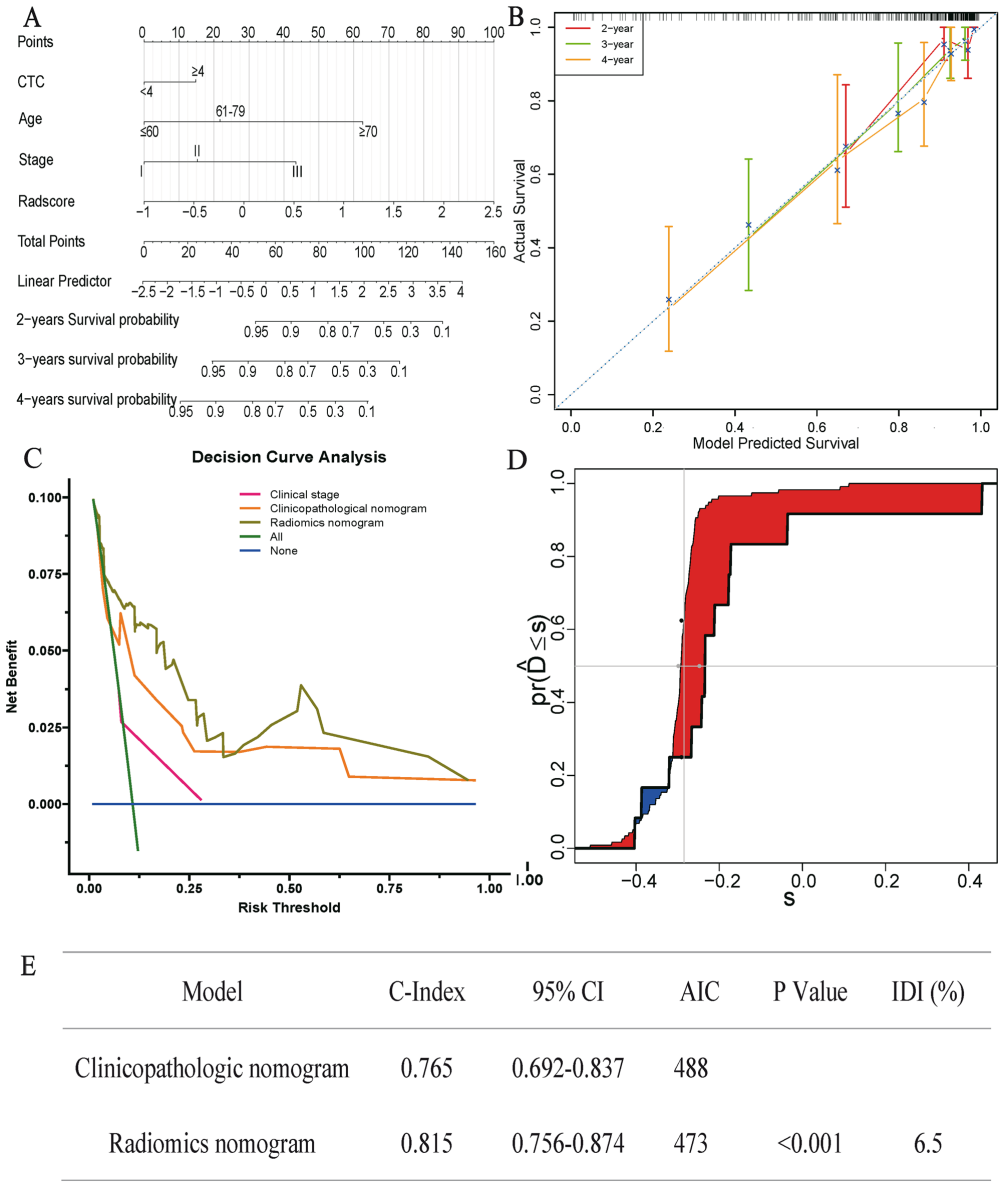
Comparison of prognostic prediction performance between radiomic nomogram and clinicopathological nomogram. **A**. The radiomics nomogram constructed using the training data set was used to predict the prognostic risk of patients with “driver gene-negative” LUAD. **B**. The calibration curves demonstrated that the radiomics nomogram had a good prediction performance of the survival probability at 2, 3 and 4 years after diagnosis. **C**. The decision curve showed that radiomics nomogram (solid brown line) was a model with a higher net income than clinicopathological nomogram (solid orange line) under most of the given thresholds. **D**. The integrated discrimination index (IDI) indicated that the prediction performance of radiomics nomogram model was 6.5% higher than that of clinicopathological nomogram model. **E**. The estimated concordance index (C-index) and Akaike information criteria (AIC) of the radiomic nomogram and clinicopathological nomogram

The clinicopathological nomogram model demonstrated promising clinical prognosis prediction performance (C-index: 0.765; 95% CI: 0.692–0.837). After combining the optimal rad-score with independent clinical prognostic characteristics, the prediction performance of the radiomics nomogram had been significantly improved (C-index: 0.815; 95% CI: 0.756–0.874) (Supplementary Fig. 6). The radiomics nomogram is presented in Fig. 2A. The nomogram calibration curves of the survival probability at 2, 3 and 4 years after diagnosis are shown in Fig. 2B, and they indicate the degree of consistency between the predicted value of the nomogram and the actual observation results.

The decision curve analysis in Fig. 2C demonstrated that across the most reasonable threshold probability range, the overall net benefit of the radiomics nomogram was higher than that of the clinicopathological nomogram. The IDI in Fig. 2D showed that the prediction performance of radiomics nomogram model was 6.5% higher than that of clinicopathological nomogram model. Figure 2E shows the estimated C-index and AIC of the models.

**Fig. 3:**
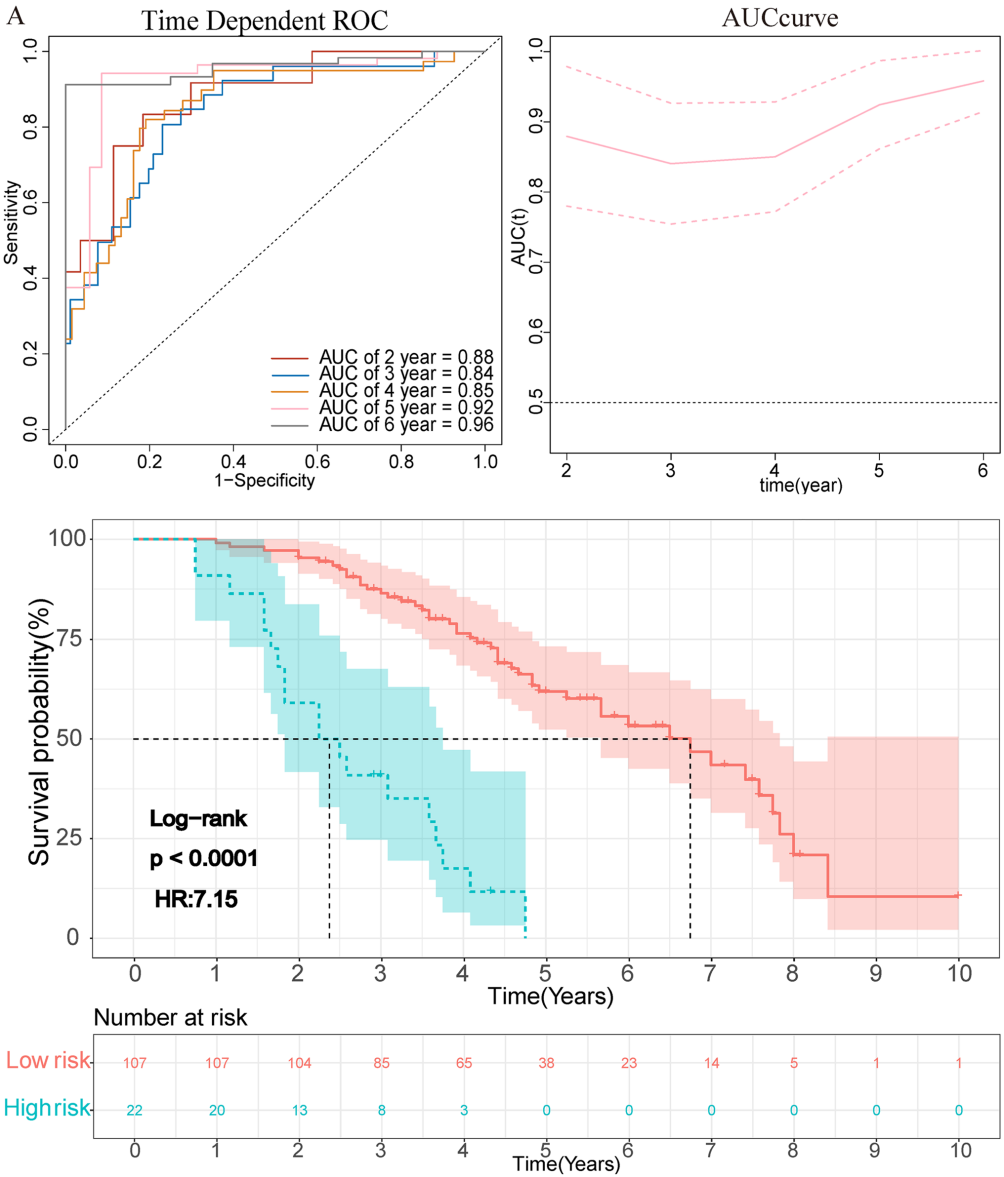

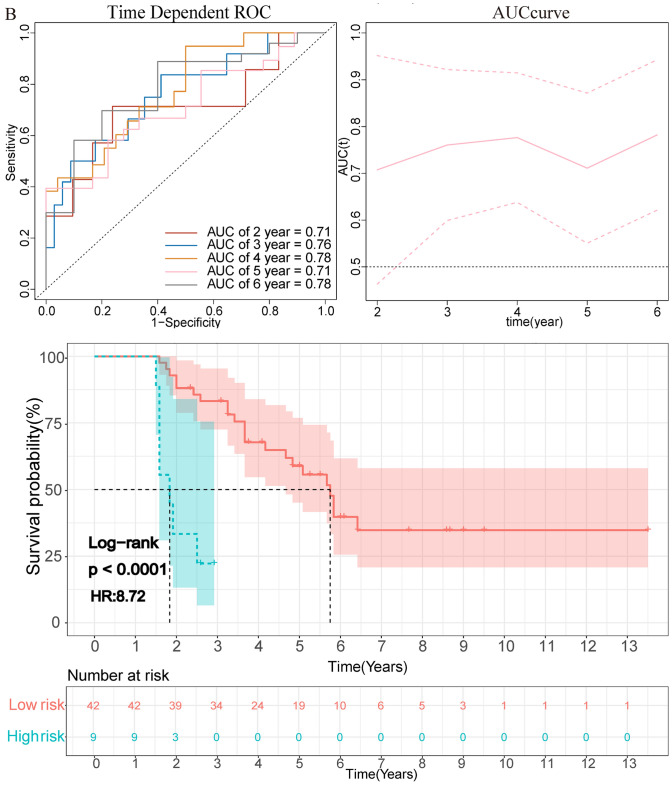
The graph showed the results of the OS curve of Kaplan–Meier survival analysis and time-dependent AUC plot in the training data set (**A**) and validation data set (**B**) after dividing the clinical prognosis score into a high-risk group and a low-risk group according to the cutoff value. The dotted line represents the corresponding median survival time

The clinical prognostic risk score of each patient according to the radiomics nomogram was calculated. The optimal cutoff risk point of 65.28 was found out by using X-tile (Supplementary Fig. 7) software and divided the patients into high-risk (> 65.28) and low-risk (≤ 65.28) groups. The prognostic score was significantly correlated with OS in the training data set (*p* < 0.001, HR = 7.15 (3.93–12.99)), and it was verified in the validation data set (*p* < 0.001, HR = 8.72 (3.00–25.30)) (Fig. 3). Patients with lower-risk score had better OS. AUROC of training set was 0.88 for 2 years and 0.85 for 4 years. Among validation set, the AUROC was 0.71 for 2 years and 0.78 for 4 years (Fig. 3).

### Genetic studies of the radiomics nomogram

**Fig. 4:**
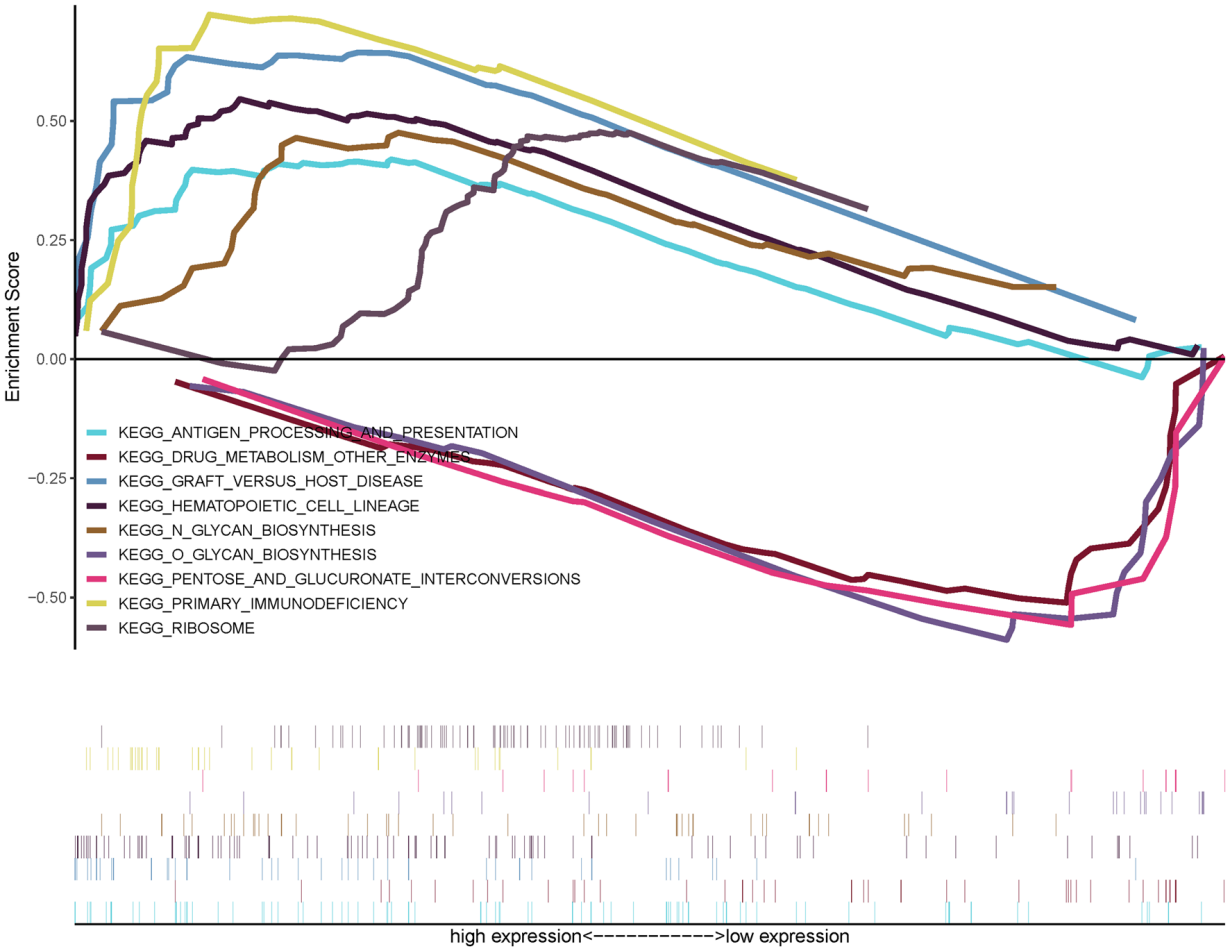
GSEA analysis for prediction model most significantly enriched pathways with enrichment scores was shown in different colors. Above the abscissa were pathways enriched in the low-risk score group, below the abscissa were pathways enriched in the high-risk score group

We explored the biological basis of the radiomics model through a GSEA analysis based on the RNA expression data of 27 LUAD patients. Available from GSEA results, there were 3 significantly enriched pathways in low-risk score group and 6 in high-risk score group (*p* < 0.05) (Supplementary Fig. 8). The eight pathways of high-risk score group (primary immunodeficiency, graft versus host disease, antigen processing and presentation, N-glycan biosynthesis, ribosome and hematopoietic cell lineage) and top three pathways of low-risk group (pentose and glucuronate interconversions, O-glycan biosynthesis and drug metabolism other enzymes) are shown in Fig. 4. The pathways suggested the biological basis for the role of the radiomics model may be due to changes in immune and metabolism-related pathways.

## Discussion

In this study, the individualized nomogram based on radiomics characteristics, age, staging and CTC count showed promising prediction efficacy of survival in patients with stage I–III “driver gene-negative” LUAD, with C-index of 0.815 (95% CI: 0.756–0.874). GSEA results showed that the low-risk group was directly related to amino acid metabolism, and the high-risk score group was related to immune and autophagy pathways.

Previous studies had shown that CTCs count was an independent prognostic factor of NSCLC [33], especially in advanced NSCLC [34, 35]. However, there were few studies on the correlation between the prognosis of “driver gene-negative” LUAD after operation and the count of CTCs. Our research confirms that CTC was an independent factor of OS in this genetically unique subset of patients. It can not only predict the prognosis before treatment, but also help monitor the dynamic response of patients during treatment [36] and changes in survival probability [37].

In this study, we used the prognostic nomogram of pre-treatment imaging features and clinical data to stratify the risk of patients with “driver gene-negative” LUAD after surgery. As the first study to use radiomics features in the survival assessment of this genetically unique subset of patients, we found that the combination of radiomics and clinical features has stronger predictive power than a single radiomics feature or clinical feature nomogram, with a higher C-index and better calibration. The decision curve analysis showed that within the most reasonable threshold probability range, the radiomics model performs better than the clinicopathological model. In this study, we focused on patients with “driver gene-negative” LUAD, which was a relatively rare subtype of NSCLC, and mutation-negative patients have limited treatment options and poor prognosis. We developed an imaging–clinical comprehensive prognostic model and conducted preliminary exploration of related pathways in order to guide individual postoperative care for those patients, especially high-risk patients.

The GSEA results showed the low-risk group was directly linked to metabolism-related pathways and the high-risk group correlated with immune-related pathways. Previous studies have also shown that amino metabolism-related pathways [38] play an important role in the development of LUAD. In addition, in this study we found that the high-risk group was related to immune and autophagy pathways, which may indicate that immunotherapy may play a therapeutic role in this population. Thus, these findings provide new treatment orientation for “driver gene-negative” postoperative patients who cannot undergo targeted therapy.

There were also some limitations in our research. First, the inevitable selection bias in patient screening and the small size data set in this study not only greatly influence on the robustness of the radiomics model but also limit the generality of our model. The sample size needs to be expanded to make the results more convincing, and the results require further independent external validation before widespread implementation in clinical practice. Second, despite the promising findings of the present study, the results cannot be generalized to other populations because gene mutation rate and other clinical factors such as smoking can be affected by race. Third, our research mainly focused on patients with LUAD and ignored other histological subtypes of lung cancer patients. Finally, gene test and some of the CTC tests were performed retrospectively; thus, the research results will inevitably be biased due to the preservation duration of the study samples.

## Conclusions

The radiomics nomogram was promising to be used as a biomarker for risk stratification for OS in patients with “driver gene-negative” LUAD. The radiomics nomogram well demonstrated the incremental value to the traditional staging system and other clinical–pathological risk factors for individualized OS estimation. Furthermore, the subsequent gene enrichment analysis can not only explain the mechanism of prognosis difference, but more importantly to provide auxiliary evidence for guiding the postoperative treatment plan for this genetically unique subset of patients.

## Supplementary Information

Below is the link to the electronic supplementary material.

Supplementary file1 (DOCX 21 KB)

Supplementary file2 (TIF 1653 KB)

Supplementary file3 (TIF 478 KB)

Supplementary file4 (TIF 1017 KB)

Supplementary file5 (TIF 628 KB)

Supplementary file6 (TIF 10800 KB)

Supplementary file7 (TIF 2063 KB)

Supplementary file8 (TIF 3016 KB)

Supplementary file9 (TIF 5755 KB)

Supplementary file10 (DOCX 50 KB)

## Abbreviations

AIC: Akaike information criteria
AUROC: Areas under the receiver operating characteristic curve
C-index: Concordance index
CT: Computed tomography
CTC: Circulating tumor cell
GEO: Gene Expression Omnibus
GSEA: Gene set enrichment analysis
ICC: Intraclass correlation coefficient
ICIs: Immune checkpoint inhibitors
IDI: Integrated discrimination improvement
LASSO: Least Absolute Shrinkage and Selection Operator
LOG: Laplacian of Gaussian
LUAD: Lung adenocarcinoma
OS: Overall survival
PD-1: Programmed death-1
PD-L1: Programmed death ligand-1
Rad-score: Radiomics score
TPS: Tumor proportion scores
