# Supplementary material for: A radiomics nomogram prediction for survival of patients with “driver gene-negative” lung adenocarcinomas (LUAD)
Source: Radiol Med. 2023 May 23;128(6):714–25. doi: 10.1007/s11547-023-01643-4 (PMC10264479; doi:10.1007/s11547-023-01643-4)
Supplement: Supplementary file 1 — Supplementary file1 (DOCX 21 KB) [file 11547_2023_1643_MOESM1_ESM.docx]

**Supplementary material E1: CT Scan Protocol**

All patients underwent contrast-enhanced CT of the chest about a week before the operation performed by using multidetector CT scanners (Aquilion 64, Canon Medical Systems, Otawara, Japan) during inspiration. Scan parameters: tube voltage of 120 kVp; maximum of 500 mA with automatic tube current modulation; field of view (FOV), 350 mm; image matrix, 512×512. Axial thin-section CT images of the whole lung were reconstructed with a section thickness of 1.0 mm using a high-resolution algorithm without interval. Iopromide (300 mg I/m1, Schering Pharmaceutical Ltd) was used as the contrast agent for enhanced scanning protocol. When 80-100 ml Iopromide was injected at 3-4 ml/s flow rate after routine nonenhanced CT, contrast enhanced scanning protocol would be applied after 60-70s. In order to ensure the uniformity of image features and to avoid feature extraction bias, patients with imaging thickness other than 1mm were excluded in our study.

**Supplementary material E2: Extracted radiomics feature**

PyRadiomics is an open-source platform in Python. The platform contains five feature classes: a class for first-order statistics, a class for shape descriptors, and texture classes gray level cooccurrence matrix^1^, gray level run length matrix^2, 3^, and gray level size zone matrix^4^. All statistic and texture classes can be used for feature extraction from both filtered and unfiltered images. Shape descriptors were independent from intensity values and therefore can only be extracted from unfiltered images. Feature extraction was supported for both single slice (2D) and whole volume (3D) segmentations^5^.

PyRadiomics can extract 14 shape features from unfiltered images, and 19 first-order features, 28 GLCM features, 16 GLRLM features, and 16 GLSZM features from each raw (unfiltered) and filtered images. Features names and mathematical formulas were provided in Table S1.

In addition, features can also be extracted from the wavelet and Laplacian of Gaussian (LoG) filtered images. For the wavelet-filter, the stationary wavelet transform implemented in the PyWavelet package was applied using the "coif1" (coiflet-1) wavelet function. Each image was filtered using either a high band-pass filter or low bandpass filter in x, y and z directions, yielding 8 different combinations of decompositions. For LoG, the image is filtered using the 3D LoG filter implemented in SimpleITK and the sigma values were changed to 0.5, 1.0, 1.5, 2.0, 3.0, 5.0mm, resulting in another 6 derived images^6^. Finally, a total of 1409 features were extracted.

**Supplementary material E3: The Packages of R Software Used for Statistical Analysis**

Made use of the "caret" package to remove highly correlated features and z-score standardization, and used "glmnet" to perform LASSO Cox regression. Survival analysis was done through the "survival" and “survminer” package. The "rms" package was applied to the establishment of multivariate Cox regression, the generation of nomograms, and the calculation of calibration curves. The comparison between the two models was done via the "stats" package. The calculation of the IDI was performed by the "survIDINRI" package. The clinical decision curve was generated by the "ggDCA" package. The ROC analysis was performed by the "pROC", "survivalROC" and " timeROC " packages. All statistical tests were two-tailed, and p <0.05 was considered statistically different.

**Supplementary material E4. Calculation formula for radiomics signature**

Radiomics score = 0.182025692 × wavelet_LLL_ngtdm_Coarseness + 0.151871358 × log_sigma_0_5_mm_3D_ngtdm_Busyness + 0.04527581 × wavelet_HLL_glszm_LargeAreaHighGrayLevelEmphasis_CE + 0.076630881 × log_sigma_0_5_mm_3D_glszm_LargeAreaHighGrayLevelEmphasis_CE + 0.089315938 × log_sigma_1_0_mm_3D_glszm_LargeAreaLowGrayLevelEmphasis_CE + 0.17942569 × log_sigma_3_0_mm_3D_ngtdm_Coarseness_CE - 0.198388867 × original_gldm_LargeDependenceHighGrayLevelEmphasis_CE

**Supplementary Figure 1.** The flowchart of the selection of “driver gene-negative” LUAD patients, including inclusion and exclusion criteria. **Step 1**, Selected 784 pathology-confirmed LUAD patients who were EGFR-mutant negative from the First Affiliated Hospital of Sun Yat-Sen University. **Step2**, FFPE tissues taken from these patients with an NGS platform using a 13-gene panel (including EGFR, KRAS, BRAF, PIK3CA, NRAS, HER2, MET, AKT1, c-KIT, PDGFRA, ALK, RET and ROS1) to determine their driver gene profile and validated using ARMS PCR for EGFR and KRAS and a FISH assay for ALK. **Step3**, the validated LUAD patients negative for EGFR, KRAS, BRAF, HER2, MET, ALK, RET and ROS1 were identified as “driver-gene-negative”. And then, randomly selected 60 pairs of fresh tumor and adjacent normal tissues from the remaining 371 patients and were used for genome-wide microarray assay to screen candidate genes followed by Western blotting and qPCR. The 60 pairs samples must meet the following criteria: 15 pairs of tissues with stage I, 15 pairs with stage II, 15 pairs with stage III, and 15 pairs with stage IV. **Step4**, filtering the target data by exclusion criteria. The final patient cohort was 180 patients and 28 patients’ fresh tumors and adjacent normal tissues sequencing results were available. **Step5**, the training and validation datasets were assigned 7-to-3, the latter included the 28 patients with available genetic data. FFPE, formalin fixed, paraffin embedded; NGS, next generation sequencing; EGFR, epidermal growth factor receptor; KRAS, kirsten rat sarcoma viral oncogene; ALK, anaplastic lymphoma kinase; ARMS, amplification refractory mutation system; PCR, polymerase chain reaction; FISH, fluorescence in situ hybridization.

**Supplementary Figure 2.** Intraclass Correlation Coefficient (ICC) distribution of radiomics features in manual segmentations by 2 radiologists. A. Histogram of calculated separately for all radiomics features. B. The stability of radiomics classification features.

**Supplementary Figure 3.** Use the least absolute contraction and selection operator (LASSO) cox regression model to screen the radiomics characteristics. The Lasso-cox regression model for 10-fold cross-validation tried to select the lowest standard tuning parameter (λ). The binomial deviation (y-axis) was plotted against the logarithm (λ) (x-axis). The binomial deviation (y-axis) was plotted against the logarithm (λ) (x-axis), and a vertical line was drawn at the best value of the minimum criteria and 1 standard error (estimated value) of the minimum criteria. A. The optimum value of λ was 0.140, and the corresponding value of log(λ)=−1.964. B. The LASSO regression coefficients of the three radiomics features sets. The vertical lines correspond to their respective best log(λ), and finally each group got 7 non-zero coefficient features.

**Supplementary Figure 4.** The 7 non-zero coefficient optimal features bar graphs. CE: contrast enhancement thin-slice chest CT images.

**Supplementary Figure 5.** The box-and-whiskers dot plots of Rad-scores for each patient. The lower and upper borders of the box represent the lower and upper quartiles (25th percentile and 75th percentile). The middle horizontal line represents the median. The lower and upper whiskers represent the minimum and maximum values of non-outliers. Extra dots represent outliers.

**Supplementary Figure 6.** The constitution of the 2 prediction models demonstrated as a forest plot.

**Supplementary Figure 7.** The X-tile plot of the prognostic score calculated by the radiomics nomogram in the training data set. A. The Supplementary Figure howed that the segmentation threshold was 65.3. B. The colorbar in the lower left corner of the figure represented the associated strength of each partition, ranging from black to bright red or green. Red indicated that the prognostic score and survival rate were inversely correlated. The x-axis from left to right represented all potential cut-off points defining the low subset from low to high, and the y-axis from top to bottom represented the cut-off points defining the high subset from high to low. C. The histogram of prognostic score.

**Supplementary Figure 8.** Separate curve of pathway enrichment results of GSEA. A. Antigen Processing and Presentation, ES 0.42, NES 1.56; B. Graft Versus Host Disease, ES 0.64, NES 1.62; C. Hematopoietic Cell Lineage, ES 0.55, NES 1.57; D. N-Glycan Biosynthesis, ES 0.47, NES 1.60; E. Primary Immunodeficiency, ES 0.72, NES 1.75; F. Ribosome, ES 0.48, NES 1.58. G. Drug Metabolism Other Enzymes, ES -0.54, NES -1.59; H. O-Glycan Biosynthesis, ES -0.62, NES -1.56; I. Pentose and Glucuronate Interconversions, ES -0.62, NES -1.56.

**References**

1. Haralick, R.M., K. Shanmugam, and I. Dinstein, Textural Features for Image Classification. Studies in Media and Communication, 1973; SMC-3(6): 610-621. doi: 10.1109/TSMC.1973.4309314.

2. Galloway, M., Texture analysis using gray level run lengths. Computer Graphics & Image Processing, 1975; 4(2): 172-179. doi: 10.1016/S0146-664X(75)80008-6.

3. Chu, A., C.M. Sehgal, and J.F. Greenleaf, Use of gray value distribution of run lengths for texture analysis. Pattern Recognition Letters, 1990; 11(6): 415-419. doi: 10.1016/0167-8655(90)90112-F.

4. Thibault, G., B. Fertil, C. Navarro, et al., Shape and texture indexes - Application to cell nuclei classification. International Journal of Pattern Recognition & Artificial Intelligence, 2013; 27(1): 1545-15. doi: 10.1142/S0218001413570024.

5. Griethuysen, J., A. Fedorov, C. Parmar, et al., Computational Radiomics System to Decode the Radiographic Phenotype. Cancer Research, 2017; 77(21): e104-e107. doi: 10.1158/0008-5472.CAN-17-0339.

6. van Griethuysen, J.J.M., A. Fedorov, C. Parmar, et al., Computational Radiomics System to Decode the Radiographic Phenotype. Cancer Res, 2017; 77(21): e104-e107. doi: 10.1158/0008-5472.Can-17-0339.
