# Supplementary figures and images for: A radiomics nomogram prediction for survival of patients with “driver gene-negative” lung adenocarcinomas (LUAD)
Source: Radiol Med. 2023 May 23;128(6):714–25. doi: 10.1007/s11547-023-01643-4 (PMC10264479; doi:10.1007/s11547-023-01643-4)

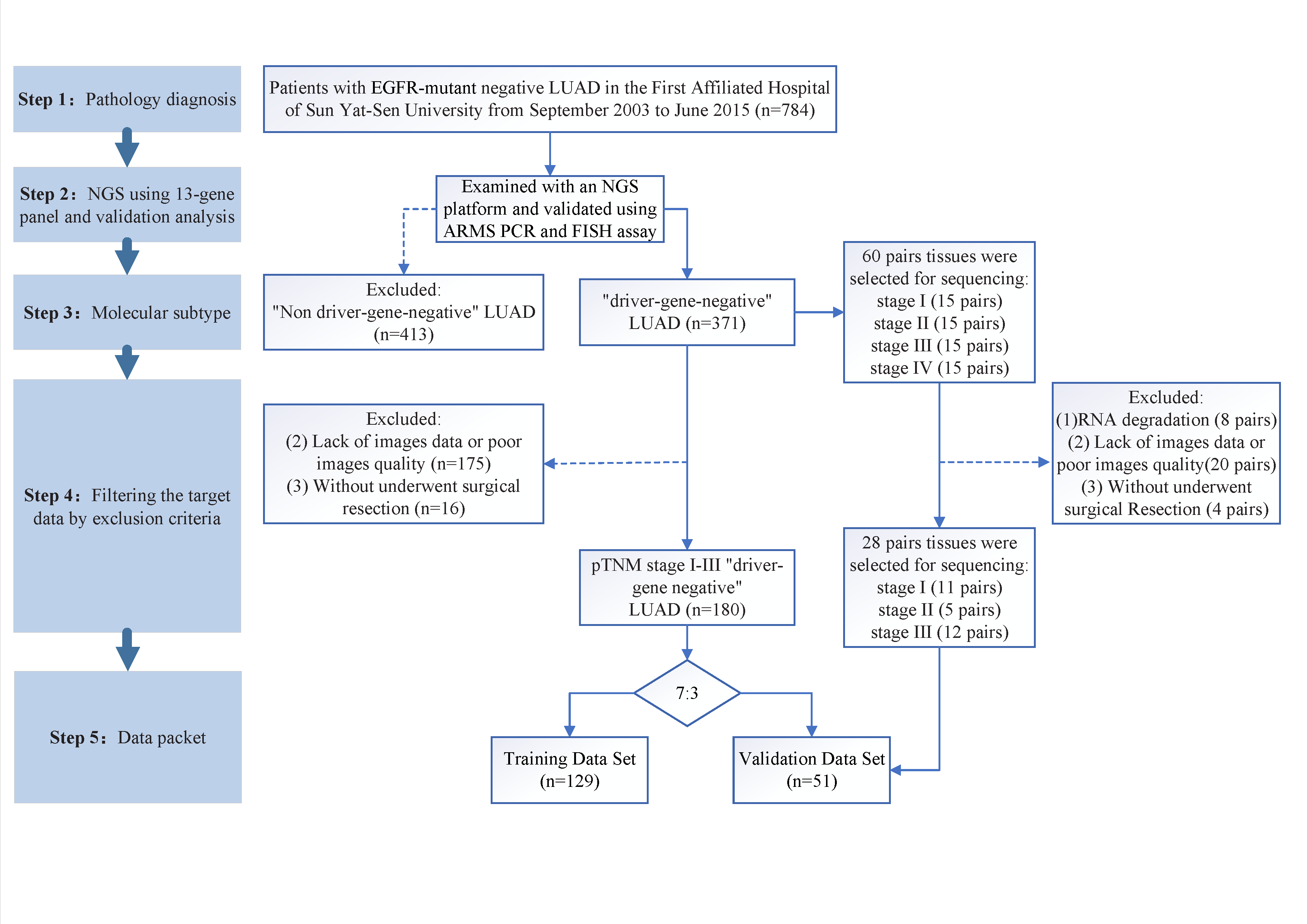

Supplement: Supplementary file 2 — Supplementary file2 (TIF 1653 KB) [file 11547_2023_1643_MOESM2_ESM.tif]

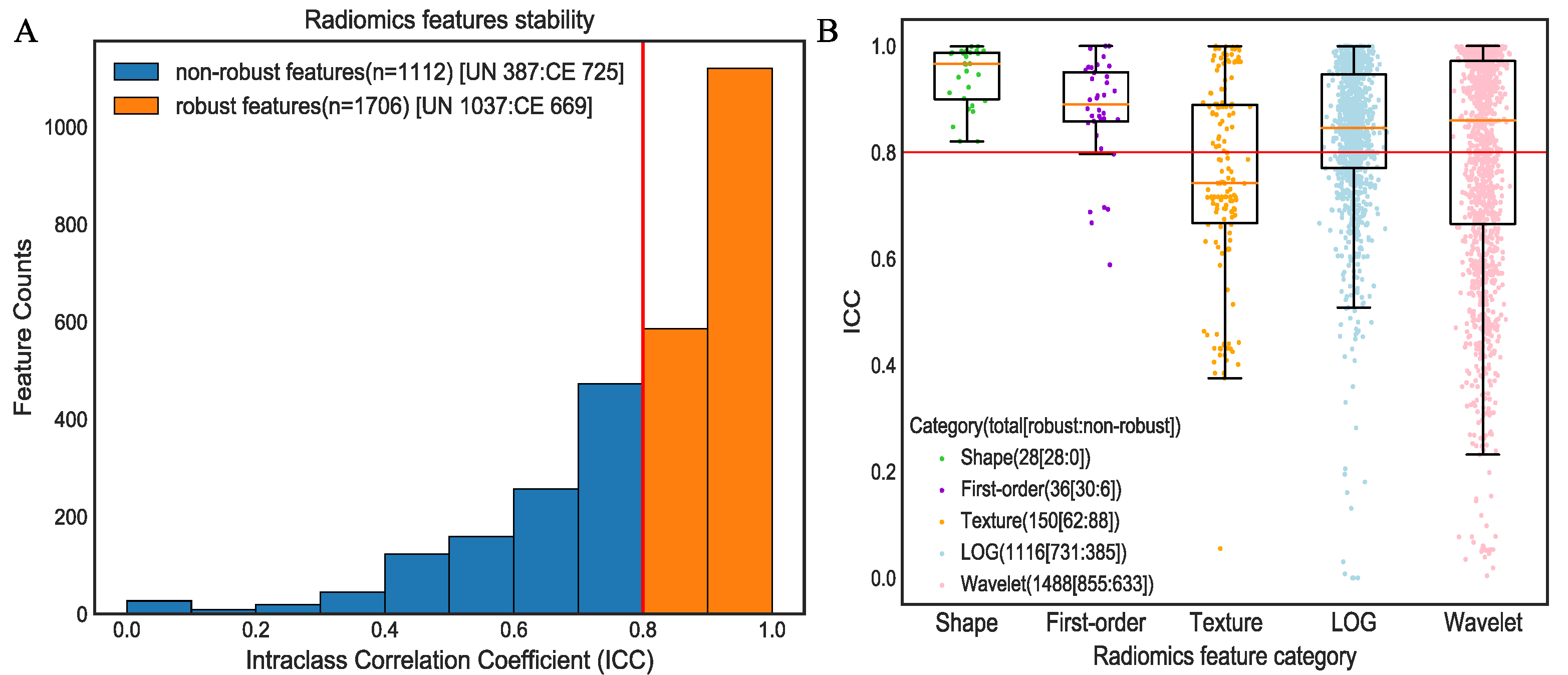

Supplement: Supplementary file 3 — Supplementary file3 (TIF 478 KB) [file 11547_2023_1643_MOESM3_ESM.tif]

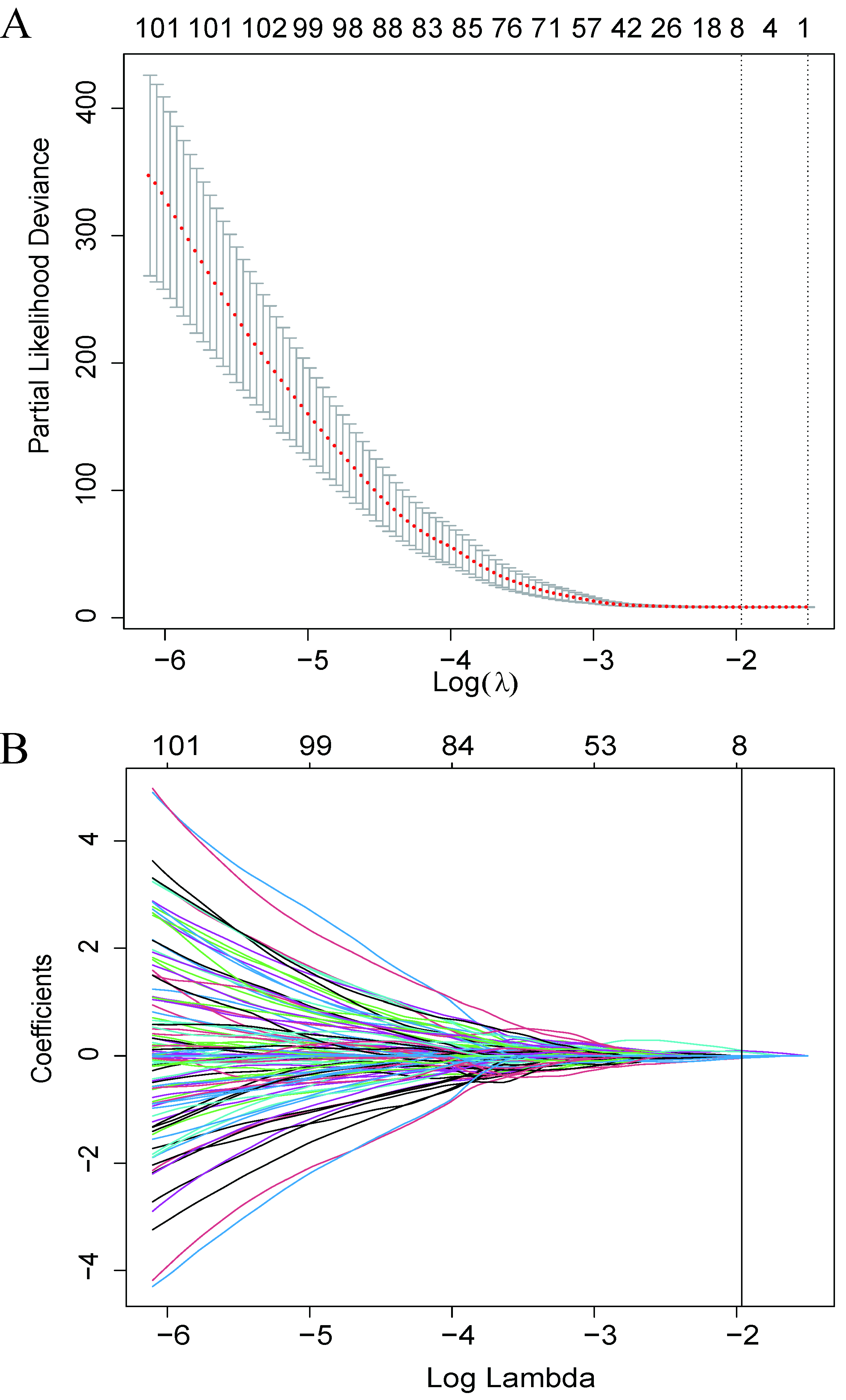

Supplement: Supplementary file 4 — Supplementary file4 (TIF 1017 KB) [file 11547_2023_1643_MOESM4_ESM.tif]

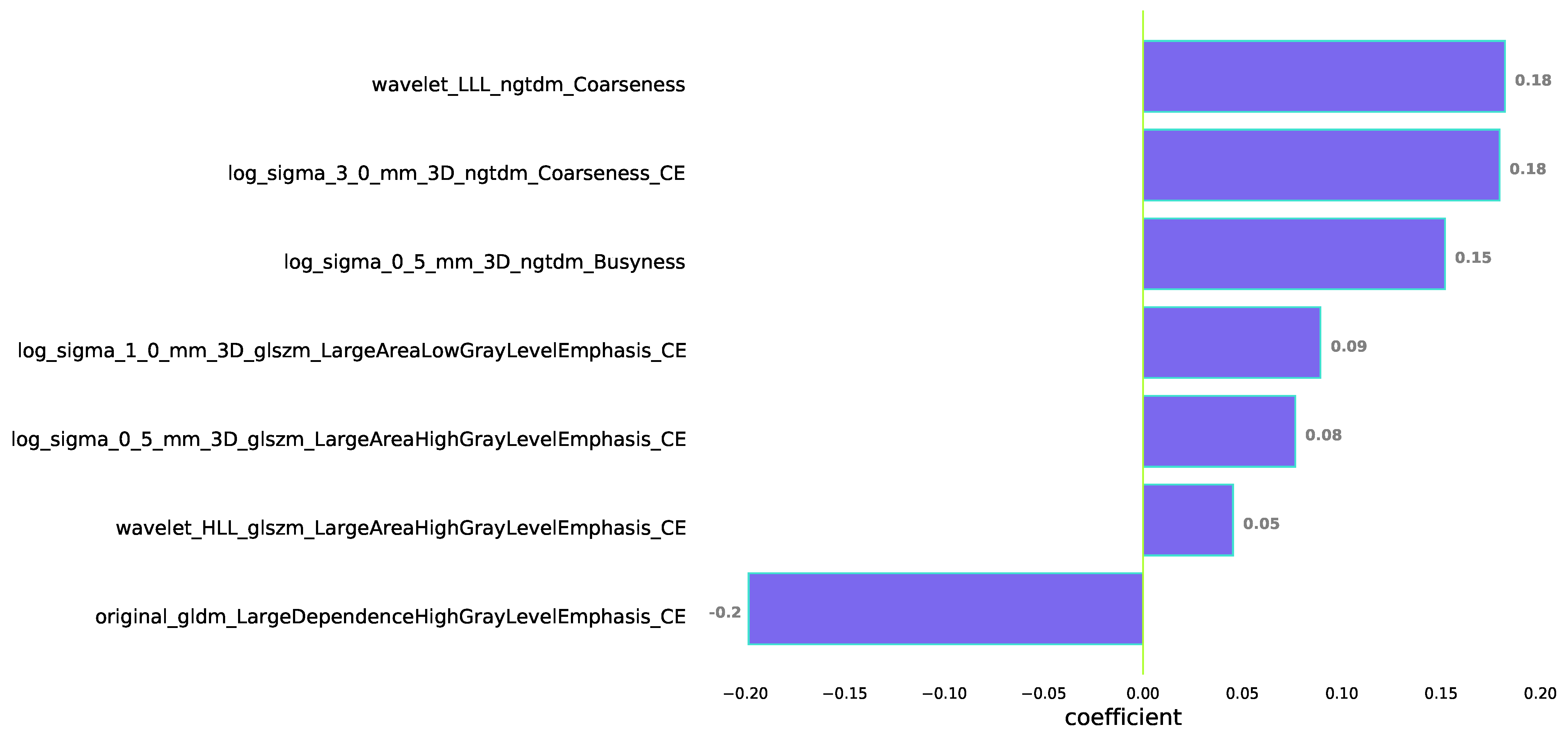

Supplement: Supplementary file 5 — Supplementary file5 (TIF 628 KB) [file 11547_2023_1643_MOESM5_ESM.tif]

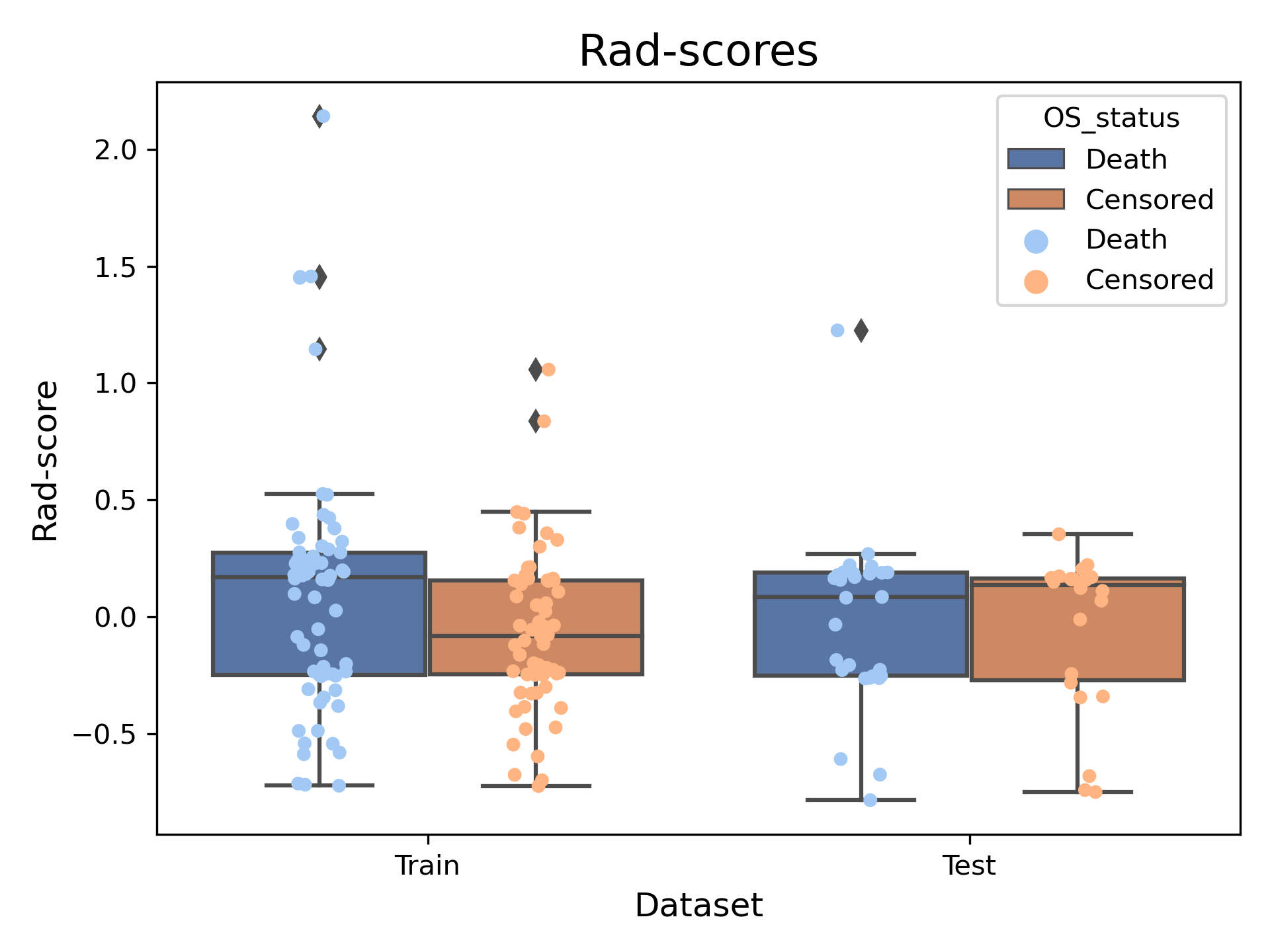

Supplement: Supplementary file 6 — Supplementary file6 (TIF 10800 KB) [file 11547_2023_1643_MOESM6_ESM.tif]

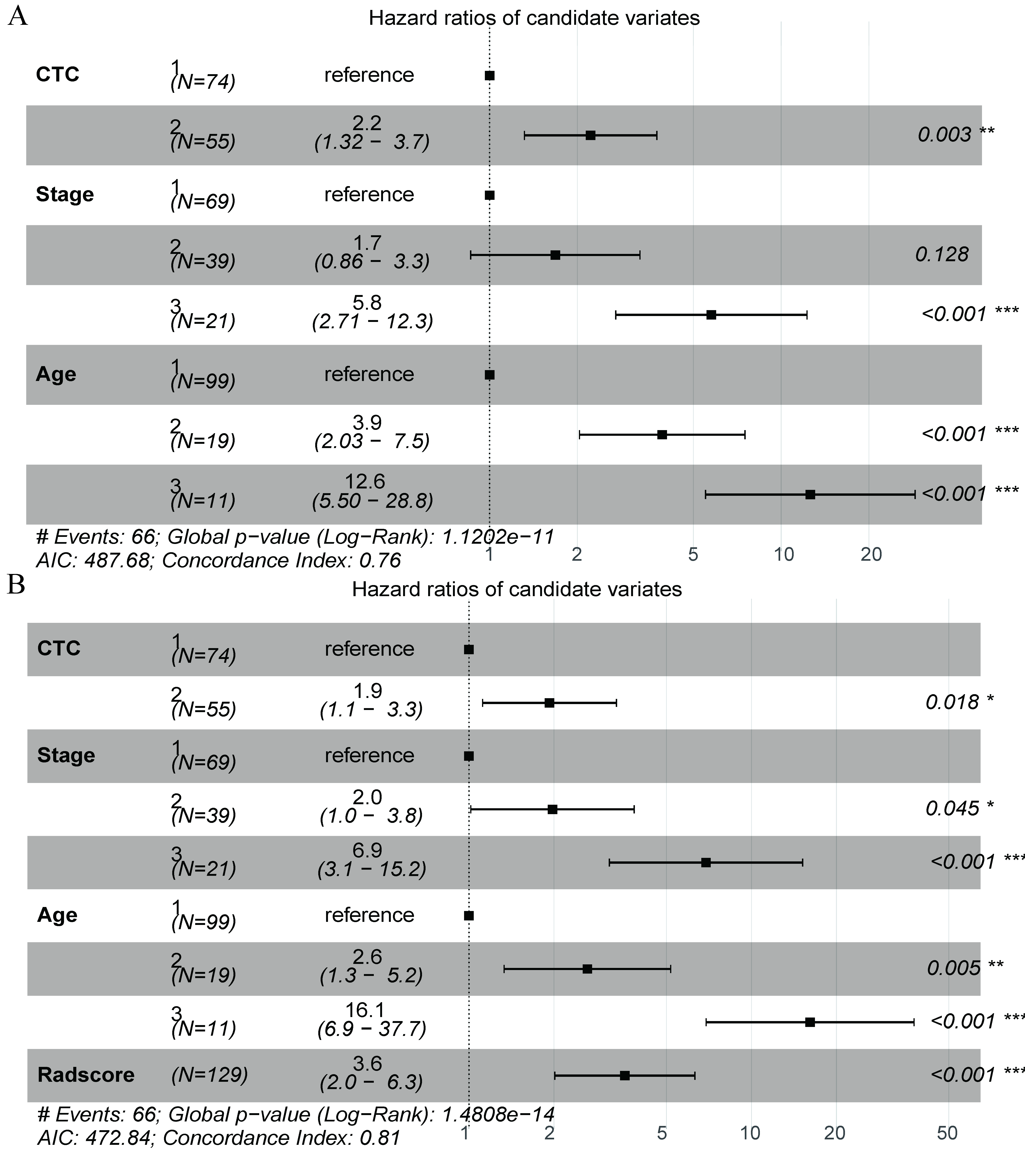

Supplement: Supplementary file 7 — Supplementary file7 (TIF 2063 KB) [file 11547_2023_1643_MOESM7_ESM.tif]

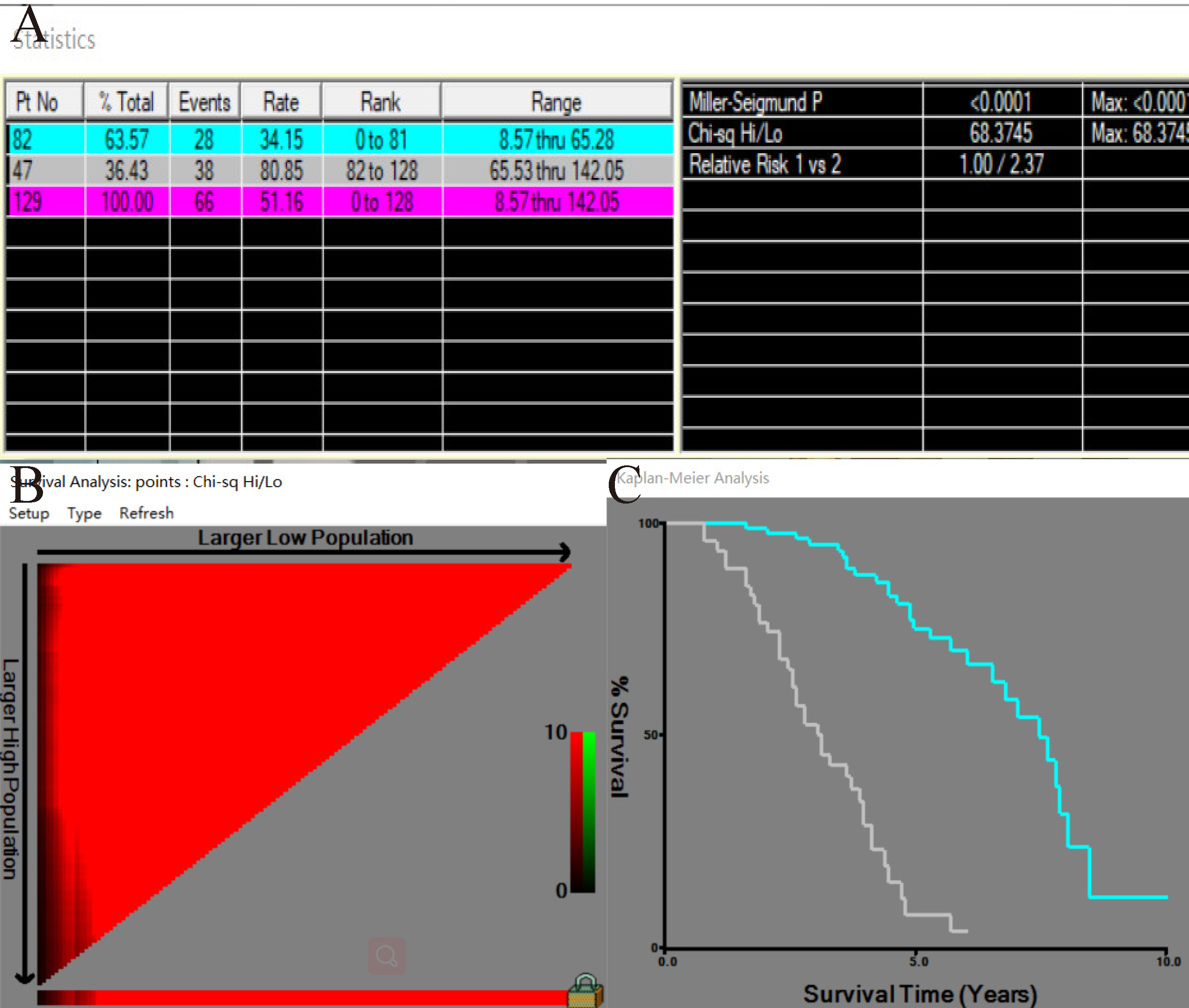

Supplement: Supplementary file 8 — Supplementary file8 (TIF 3016 KB) [file 11547_2023_1643_MOESM8_ESM.tif]

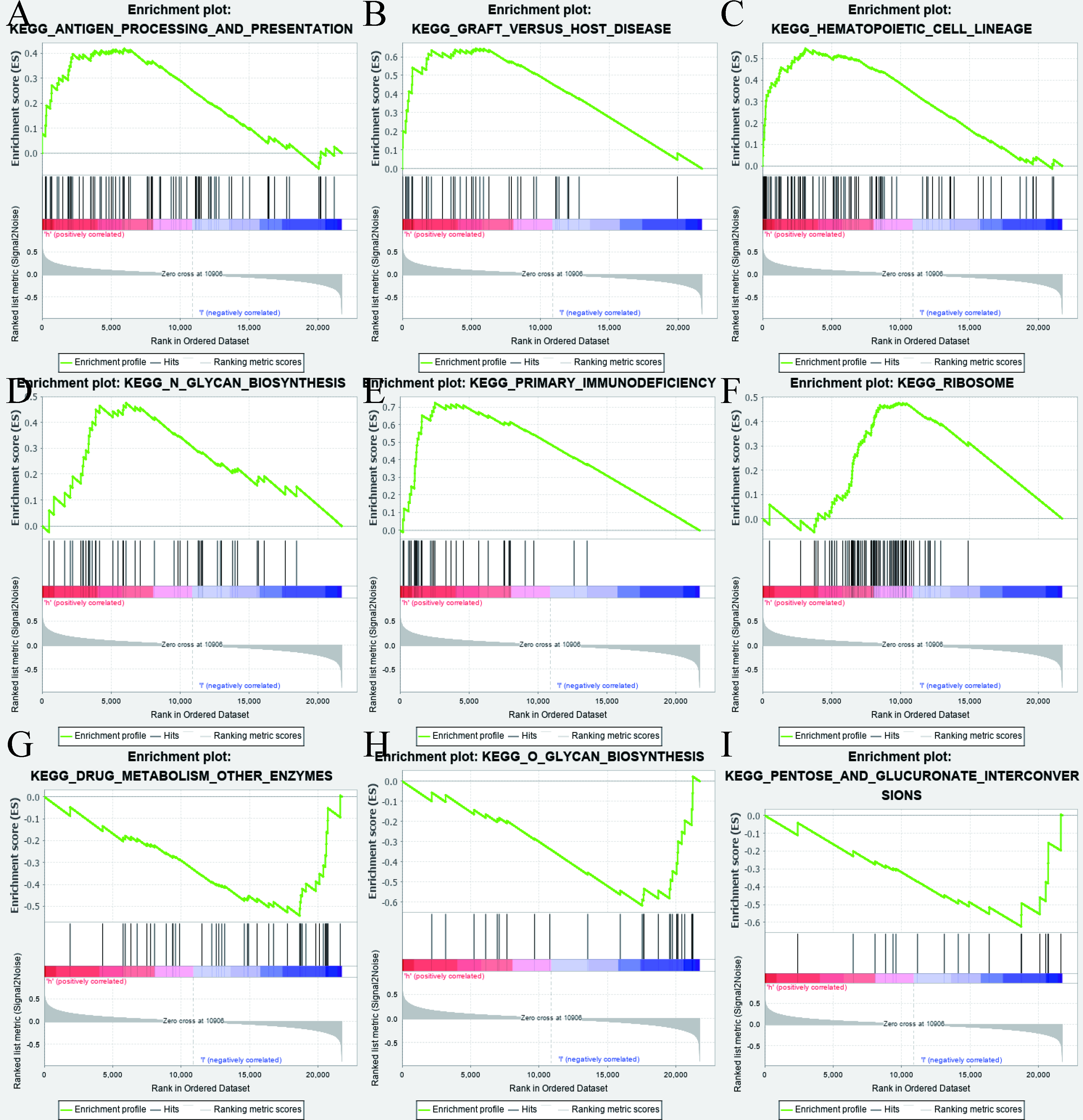

Supplement: Supplementary file 9 — Supplementary file9 (TIF 5755 KB) [file 11547_2023_1643_MOESM9_ESM.tif]
