# Supplementary material for: A radiomics nomogram prediction for survival of patients with “driver gene-negative” lung adenocarcinomas (LUAD)
Source: Radiol Med. 2023 May 23;128(6):714–25. doi: 10.1007/s11547-023-01643-4 (PMC10264479; doi:10.1007/s11547-023-01643-4)
Supplement: Supplementary file 10 — Supplementary file10 (DOCX 50 KB) [file 11547_2023_1643_MOESM10_ESM.docx]

**Table S1.** Definition of extracted radiomics features^1^

| **Parameter** | | | **Formula** | **Description** |
| --- | --- | --- | --- | --- |
| **First-order Statistics** (19 features)  ⚫XX be a set of N_p_ voxels included in the ROI  ⚫P(i) be the first order histogram with N_g_ discrete intensity levels, where N_g_ is the number of non-zero bins, equally spaced from 0 with a width defined in the **binWidth** parameter.  ⚫p(i) be the normalized first order histogram and equal to $\frac{P(i)}{N_{p}}$ | | **Energy** | $energy=\sum_{i=1}^{N_{p}} {(X\left( i \right)+c)}^{2}$  Here, c is optional value, defined by **voxelArrayShift**, which shifts the intensities to prevent negative values in X | Energy is a measure of the magnitude of voxel values in an image. |
|  |  | **Total Energy** | $total energy=V_{voxel}\sum_{i=1}^{N_{p}} {(X\left( i \right)+c)}^{2}$ | Total Energy is the value of Energy feature scaled by the volume of the voxel in cubic mm. |
|  |  | **Entropy** | $entropy=-\sum_{i=1}^{N_{g}} {p(i)log}_{2}(p\left( i \right)+\epsilon)$  Here, ϵ is an arbitrarily small positive number (≈2.2×10^-16^). | Entropy specifies the uncertainty/randomness in the image values. |
|  |  | **Minimum, Maximum** | minimum=min(X), maximum=max(X) |  |
|  |  | **10th/90th percentile** |  | The 10^th^ /90^th^ percentile of X |
|  |  | **Mean** | $mean=\frac{1}{N_{p}}\sum_{i=1}^{N_{p}} X\left( i \right)$ | The average gray level intensity within the ROI. |
|  |  | **Median** |  | The median gray level intensity within the ROI. |
|  |  | **Interquartile Range** | interquartile range=P_75_-P_25_  Here P_25_ and P_75_ are the 25^th^ and 75^th^ percentile of the image array, respectively. |  |
|  |  | **Range** | range=max(X)-min(X) | The range of gray values in the ROI. |
|  |  | **Mean Absolute Deviation (MAD)** | $MAD=\frac{1}{N_{p}}\sum_{i=1}^{N_{p}} \vert X\left( i \right)-\bar{X}\vert$ |  |
|  |  | **Robust Mean Absolute Deviation (rMAD)** | $rMAD=\frac{1}{N_{10-90}}\sum_{i=1}^{N_{10-90}} \vert X_{10-90}\left( i \right)-\bar{X}_{10-90}\vert$ | Robust Mean Absolute Deviation is the mean distance of all intensity values from the Mean Value calculated on the subset of image array with gray levels in between, or equal to the 10^th^ and 90^th^ percentile. |
|  |  | **Root Mean Squared (RMS)** | $RMS=\sqrt{\frac{1}{N_{p}}\sum_{i=1}^{N_{p}} {(X\left( i \right)+c)}^{2}}$ | RMS is the square-root of the mean of all the squared intensity values. |
|  |  | **Standard Deviation** | $standard deviation=\sqrt{\frac{1}{N_{p}}\sum_{i=1}^{N_{p}} {(X\left( i \right)-\bar{X})}^{2}}$ | tandard Deviation measures the amount of variation or dispersion from the Mean Value. |
|  |  | **Skewness** | $skewness=\frac{\mu_{3}}{\sigma^{3}}=\frac{\frac{1}{N_{p}}\sum_{i=1}^{N_{p}} {(X\left( i \right)-\bar{X})}^{3}}{\left( \sqrt{\frac{1}{N_{p}}\sum_{i=1}^{N_{p}} {(X\left( i \right)-\bar{X})}^{2}} \right)^{3}}$  Where $\mu_{3}$ is the 3^rd^ central moment. | Skewness measures the asymmetry of the distribution of values about the Mean value. |
|  |  | **Kurtosis** | $kurtosis=\frac{\mu_{4}}{\sigma^{4}}=\frac{\frac{1}{N_{p}}\sum_{i=1}^{N_{p}} {(X\left( i \right)-\bar{X})}^{4}}{\left( \frac{1}{N_{p}}\sum_{i=1}^{N_{p}} {(X\left( i \right)-\bar{X})}^{2} \right)^{4}}$  Where $\mu_{4}$ is the 4^th^ central moment. | Kurtosis is a measure of the ‘peakedness’ of the distribution of values in the image ROI. |
|  |  | **Variance** | $variance=\frac{1}{N_{p}}\sum_{i=1}^{N_{p}} {(X\left( i \right)-\bar{X})}^{2}$ | Variance is the the mean of the squared distances of each intensity value from the Mean value. |
|  |  | **Uniformity** | $uniformity=\sum_{i=1}^{N_{g}} {p\left( i \right)}^{2}$ | Uniformity is a measure of the sum of the squares of each intensity value. |
| **Shape** (14 features)  ⚫$N_{v}$ represent the number of voxels included in the ROI  ⚫$N_{f}$ represent the number of faces (triangles) defining the Mesh.  ⚫*V* the volume of the mesh in mm^3^.  ⚫*A* the surface area of the mesh in mm^2^. | | **Elongation** | $elongation=\sqrt{\frac{\lambda_{minor}}{\lambda_{major}}}$  Here, $\lambda_{major}$ and $\lambda_{minor}$ are the lengths of the largest and second largest principal component axes. |  |
|  |  | **Flatness** | $flatness=\sqrt{\frac{\lambda_{least}}{\lambda_{major}}}$ |  |
|  |  | **Least axis length** | $least axis=4\sqrt{\lambda_{least}}$ |  |
|  |  | **Major axis length** | $major axis=4\sqrt{\lambda_{major}}$ |  |
|  |  | **Maximum 2D diameter (Column)** |  | Maximum 2D diameter (Column) is defined as the largest pairwise Euclidean distance between tumor surface mesh vertices in the row-slice (usually the coronal) plane. |
|  |  | **Maximum 2D diameter (Row)** |  | Maximum 2D diameter (Row) is defined as the largest pairwise Euclidean distance between tumor surface mesh vertices in the column-slice (usually the sagittal) plane. |
|  |  | **Maximum 2D diameter (Slice)** |  | Maximum 2D diameter (Slice) is defined as the largest pairwise Euclidean distance between tumor surface mesh vertices in the row-column (generally the axial) plane. |
|  |  | **Maximum 3D diameter** |  | Maximum 3D diameter is defined as the largest pairwise Euclidean distance between tumor surface mesh vertices. |
|  |  | **Mesh volume** | $V_{i}=\frac{{Oa}_{i}\cdot\left( {Ob}_{i}\times{Oac}_{i} \right)}{6} (1)$  $V=\sum_{i=1}^{N_{f}} V_{i} (2)$ |  |
|  |  | **Minor axis length** | $minor axis=4\sqrt{\lambda_{minor}}$ |  |
|  |  | **Sphericity** | $sphericity=\frac{\sqrt[3]{36\pi V^{2}}}{A}$ | Sphericity is a measure of the roundness of the shape of the tumor region relative to a sphere |
|  |  | **Surface area** | $A_{i}=\frac{1}{2}{\vert a}_{i}b_{i}\times a_{i}c_{i}\vert(1)$  $A=\sum_{i=1}^{N_{f}} A_{i} (2)$  $a_{i}b_{i}$ and $a_{i}c_{i}$ are edges of the $i^{th}$ triangle in the mesh, formed by vertices $a_{i}$, $b_{i}$ and $c_{i}$. | To calculate the surface area, first the surface area $A_{i}$ of each triangle in the mesh is calculated (1). The total surface area is then obtained by taking the sum of all calculated sub-areas (2). |
|  |  | **Surface Area to Volume ratio** | $surface to volume ratio=\frac{A}{V}$ |  |
|  |  | **Voxel volume** | $V_{voxel}=\sum_{k=1}^{N_{v}} V_{k}$ |  |
|  |  | **Elongation** | $elongation=\sqrt{\frac{\lambda_{minor}}{\lambda_{major}}}$ |  |
| **Texture** | **Gray Level Cooccurence Matrix** (**GLCM**, 24 features)  ⚫ϵ be an arbitrarily small positive number (≈2.2×10^-16^)  ⚫$\mathbf{P}(i,j)$ be the co-occurence matrix for an arbitrary δ and θ  ⚫$p(i,j)$ be the normalized co-occurence matrix and equal to $\frac{\mathbf{P}(i,j)}{\sum\mathbf{P}(i,j)}$  ⚫$N_{g}$ be the number of discrete intensity levels in the image  ⚫$p_{x}(i)=\sum_{j=1}^{N_{g}} p(i,j)$ be the marginal row probabilities  ⚫$p_{y}(j)=\sum_{i=1}^{N_{g}} p(i,j)$ be the marginal column probabilities  $●e mean gray level intensity$  ${●\mu}_{x} be the mean gray level intensity of pxpx and defined as \mu_{x}=\sum_{i=1}^{N_{g}} p_{x}(i)i$  $●\mu_{y} be the mean gray level intensity of pxpx and defined as \mu_{y}=\sum_{j=1}^{N_{g}} p_{y}(j)j$  ⚫$\sigma_{x}$ be the standard deviation of $p_{x}$  ⚫$\sigma_{y}$ be the standard deviation of $p_{y}$  ⚫$p_{x+y}\left( k \right)=\sum_{i=1}^{N_{g}} \sum_{j=1}^{N_{g}} p\left( i,j \right),where i+j=k, and k=2,3,\ldots,2N_{g}$  ⚫$p_{x-y}\left( k \right)=\sum_{i=1}^{N_{g}} \sum_{j=1}^{N_{g}} p\left( i,j \right),where \vert i-j\vert=k, and k=0,1,\ldots,N_{g}-1$  ⚫$HX=-\sum_{i=1}^{N_{g}} p_{x}\left( i \right){log}_{2}\left( p_{x}\left( i \right)+\epsilon\right)$  $be the entropy of p_{x}$  ⚫$HX=-\sum_{j=1}^{N_{g}} p_{y}\left( j \right){log}_{2}\left( p_{y}\left( j \right)+\epsilon\right) be the entropy of p_{y}$  $●HXY=-\sum_{i=1}^{N_{g}} \sum_{j=1}^{N_{g}} p\left( i,j \right){log}_{2}\left( p\left( j \right)+\epsilon\right)$  $be the entropy of p(i,j)$  $●HXY1=-\sum_{i=1}^{N_{g}} \sum_{j=1}^{N_{g}} p\left( i,j \right){log}_{2}\left( p_{x}\left( i \right)p_{y}\left( j \right)+\epsilon\right)$  $●HXY2=-\sum_{i=1}^{N_{g}} \sum_{j=1}^{N_{g}} p_{x}\left( i \right)p_{y}\left( j \right){log}_{2}\left( p_{x}\left( i \right)p_{y}\left( j \right)+\epsilon\right)$ | **Autocorrelation** | $autocorrelation=\sum_{i=1}^{N_{g}} \sum_{j=1}^{N_{g}} p(i,j)ij$ | Autocorrelation is a measure of the magnitude of the fineness and coarseness of texture. |
|  |  | **Joint Average** | $joint average=\mu_{x}=\sum_{i=1}^{N_{g}} \sum_{j=1}^{N_{g}} p(i,j)i$ |  |
|  |  | **Cluster Prominence** | $cluster prominence=\sum_{i=1}^{N_{g}} \sum_{j=1}^{N_{g}} {(i+j-\mu_{x}-\mu_{y})}^{4}p(i,j)$ | Cluster Prominence is a measure of the skewness and asymmetry of the GLCM. |
|  |  | **Cluster Shade** | $cluster shade=\sum_{i=1}^{N_{g}} \sum_{j=1}^{N_{g}} {(i+j-\mu_{x}-\mu_{y})}^{3}p(i,j)$ | Cluster Shade is a measure of the skewness and uniformity of the GLCM. |
|  |  | **Cluster Tendency** | $cluster tendency=\sum_{i=1}^{N_{g}} \sum_{j=1}^{N_{g}} {(i+j-\mu_{x}-\mu_{y})}^{2}p(i,j)$ | Cluster Tendency is a measure of groupings of voxels with similar gray-level values. |
|  |  | **Contrast** | $contrast=\sum_{i=1}^{N_{g}} \sum_{j=1}^{N_{g}} {(i-j)}^{2}p(i,j)$ | Contrast is a measure of the local intensity variation, favoring values away from the diagonal (i=j). |
|  |  | **Correlation** | $correlation=\frac{\sum_{i=1}^{N_{g}} \sum_{j=1}^{N_{g}} p\left( i,j \right)ij-\mu_{x}\mu_{y}}{\sigma_{x}(i)\sigma_{y}(j)}$ | Correlation is a value between 0 (uncorrelated) and 1 (perfectly correlated) showing the linear dependency of gray level values to their respective voxels in the GLCM. |
|  |  | **Difference Average** | $difference average=\sum_{k=0}^{N_{g-1}} kp_{x-y}(k)$ | Difference Average measures the relationship between occurrences of pairs with similar intensity values and occurrences of pairs with differing intensity values. |
|  |  | **Difference Entropy** | $difference entropy=\sum_{k=0}^{N_{g-1}} p_{x-y}(k)\log_{2}(p_{x-y}\left( k \right)+\epsilon)$ | Difference Entropy is a measure of the randomness/variability in neighborhood intensity value differences. |
|  |  | **Difference Variance** | $difference entropy=\sum_{k=0}^{N_{g-1}} {(k-DA)}^{2}p_{x-y}\left( k \right)$ | Difference Variance is a measure of heterogeneity that places higher weights on differing intensity level pairs that deviate more from the mean. |
|  |  | **Dissimilarity** | $dissimilarity=\sum_{i=1}^{N_{g}} \sum_{j=1}^{N_{g}} \vert i-j\vert p(i,j)$ |  |
|  |  | **Joint Energy** | $joint energy=\sum_{i=1}^{N_{g}} \sum_{j=1}^{N_{g}} {(p\left( i,j \right))}^{2}$ | Energy is a measure of homogeneous patterns in the image. |
|  |  | **Joint Entropy** | $joint entropy=-\sum_{i=1}^{N_{g}} \sum_{j=1}^{N_{g}} p\left( i,j \right)\log_{2}(p\left( i,j \right)+\epsilon)$ | Joint entropy is a measure of the randomness/variability in neighborhood intensity values. |
|  |  | **Informational Measure of Correlation (IMC) 1** | $IMC 1=\frac{HXY-HXY1}{max\{HX,HY\}}$ |  |
|  |  | **Informational Measure of Correlation (IMC) 2** | $IMC 2=\sqrt{1-e^{-2(HXY2-HXY)}}$ |  |
|  |  | **Inverse Difference Moment (IDM)** | $IDM =\sum_{k=0}^{N_{g}-1} \frac{p_{x-y}\left( k \right)}{1+k^{2}}$ | IDM is a measure of the local homogeneity of an image. |
|  |  | **Maximal Correlation Coefficient (MCC)** | $MCC=\sqrt{second largest eigenvalue of Q}$  $Q(i,j)=\sum_{k=0}^{N_{g}} \frac{p\left( i,k \right)p\left( j,k \right)}{p_{x}\left( i \right)p_{y}\left( k \right)}$ | The Maximal Correlation Coefficient is a measure of complexity of the texture and 0≤MCC≤1. |
|  |  | **Inverse Difference Moment Normalized (IDMN)** | $IDMN=\sum_{k=0}^{N_{g}-1} \frac{p_{x-y}\left( k \right)}{1+(\frac{k^{2}}{N_{g}^{2}})}$ |  |
|  |  | **Inverse Difference (ID)** | $ID=\sum_{k=0}^{N_{g}-1} \frac{p_{x-y}\left( k \right)}{1+k}$ |  |
|  |  | **Inverse Difference Normalized (IDN)** | $IDN=\sum_{k=0}^{N_{g}-1} \frac{p_{x-y}\left( k \right)}{1+(\frac{k}{N_{g}})}$ |  |
|  |  | **Inverse Variance** | $inverse variance=\sum_{k=1}^{N_{g}-1} \frac{p_{x-y}\left( k \right)}{k^{2}}$ |  |
|  |  | **Maximum Probability** | $maximum probability=max(p(i,j))$ | Maximum Probability is occurrences of the most predominant pair of neighboring intensity values. |
|  |  | **Sum Average** | $sum average=\sum_{k=2}^{{2N}_{g}} p_{x+y}(k)k$ | Sum Average measures the relationship between occurrences of pairs with lower intensity values and occurrences of pairs with higher intensity values. |
|  |  | **Sum Entropy** | $sum entropy=\sum_{k=2}^{{2N}_{g}} p_{x+y}\left( k \right){log}_{2}(p_{x+y}\left( k \right)+\epsilon)$ | Sum Entropy is a sum of neighborhood intensity value differences. |
|  | **Gray Level Run Length Matrix** (**GLRLM**, 16 features)  ⚫$N_{g}$ be the number of discrete intensity values in the image  ⚫$N_{s}$ be the number of discrete zone sizes in the image  ⚫$N_{p}$ be the number of voxels in the image  ⚫$N_{z}$ be the number of zones in the ROI, which is equal to $\sum_{i=1}^{N_{g}} \sum_{j=1}^{N_{s}} \mathbf{P}(i,j)$ and 1≤$N_{z}$≤$N_{p}$  ⚫$\mathbf{P}(i,j)$ be the size zone matrix  ⚫$p\left( i,j \right)$ be the normalized size zone matrix, defined as  $p(i,j)=\frac{\mathbf{P}(i,j)}{N_{z}}$ | **Small Area Emphasis (SAE)** | $SAE= \frac{\sum_{i=1}^{N_{g}} \sum_{j=1}^{N_{s}} \frac{\mathbf{P}(i,j)}{j^{2}}}{N_{z}}$ | SAE is a measure of the distribution of small size zones. |
|  |  | **Large Area Emphasis (LAE)** | $LAE= \frac{\sum_{i=1}^{N_{g}} \sum_{j=1}^{N_{s}} \mathbf{P}(i,j)j^{2}}{N_{z}}$ | LAE is a measure of the distribution of large area size zones. |
|  |  | **Gray Level Non-Uniformity (GLN)** | $GLN= \frac{\sum_{i=1}^{N_{g}} (\sum_{j=1}^{N_{s}} \mathbf{P}(i,j))^{2}}{N_{z}}$ | GLN measures the variability of gray-level intensity values in the image. |
|  |  | **Gray Level Non-Uniformity Normalized (GLNN)** | $GLNN= \frac{\sum_{i=1}^{N_{g}} (\sum_{j=1}^{N_{s}} \mathbf{P}(i,j))^{2}}{N_{z}^{2}}$ | GLNN measures the variability of gray-level intensity values in the image. |
|  |  | **Size-Zone Non-Uniformity (SZN)** | $SZN= \frac{\sum_{i=1}^{N_{s}} (\sum_{j=1}^{N_{g}} \mathbf{P}(i,j))^{2}}{N_{z}}$ | SZN measures the variability of size zone volumes in the image. |
|  |  | **Size-Zone Non-Uniformity Normalized (SZNN)** | $SZNN= \frac{\sum_{i=1}^{N_{s}} (\sum_{j=1}^{N_{j}} \mathbf{P}(i,j))^{2}}{N_{z}^{2}}$ | SZNN measures the variability of size zone volumes throughout the image. |
|  |  | **Zone Percentage (ZP)** | $ZP=\frac{N_{z}}{N_{p}}$ | ZP measures the coarseness of the texture by taking the ratio of number of zones and number of voxels in the ROI. |
|  |  | **Gray Level Variance (GLV)** | $GLV=\sum_{i=1}^{N_{g}} \sum_{j=1}^{N_{s}} p(i,j){(i-\mu)}^{2}$  $Here, \mu=\sum_{i=1}^{N_{g}} \sum_{j=1}^{N_{s}} p(i,j)i$ | GLV measures the variance in gray level intensities for the zones. |
|  |  | **Zone Entropy (ZE)** | $ZE=\sum_{i=1}^{N_{g}} \sum_{j=1}^{N_{s}} p\left( i,j \right){log}_{2}(p\left( i,j \right)+\epsilon)$ | ZE measures the uncertainty/randomness in the distribution of zone sizes and gray levels. |
|  |  | **Zone Variance (ZV)** | $ZV=\sum_{i=1}^{N_{g}} \sum_{j=1}^{N_{s}} p(i,j){(j-\mu)}^{2}$  $Here, \mu=\sum_{i=1}^{N_{g}} \sum_{j=1}^{N_{s}} p(i,j)j$ | ZV measures the variance in zone size volumes for the zones. |
|  |  | **Low Gray Level Zone Emphasis (LGLZE)** | $LGLZE= \frac{\sum_{i=1}^{N_{g}} \sum_{j=1}^{N_{s}} \frac{\mathbf{P}(i,j)}{i^{2}}}{N_{z}}$ | LGLZE measures the distribution of lower gray-level size zones. |
|  |  | **High Gray Level Zone Emphasis (HGLZE)** | $HGLZE= \frac{\sum_{i=1}^{N_{g}} \sum_{j=1}^{N_{s}} \mathbf{P}(i,j)i^{2}}{N_{z}}$ | HGLZE measures the distribution of the higher gray-level values. |
|  |  | **Small Area Low Gray Level Emphasis (SALGLE)** | $SALGLE= \frac{\sum_{i=1}^{N_{g}} \sum_{j=1}^{N_{s}} \frac{\mathbf{P}(i,j)}{i^{2}j^{2}}}{N_{z}}$ | SALGLE measures the proportion in the image of the joint distribution of smaller size zones with lower gray-level values. |
|  |  | **Small Area High Gray Level Emphasis (SAHGLE)** | $SAHGLE= \frac{\sum_{i=1}^{N_{g}} \sum_{j=1}^{N_{s}} \frac{\mathbf{P}(i,j)i^{2}}{j^{2}}}{N_{z}}$ | SAHGLE measures the proportion in the image of the joint distribution of smaller size zones with higher gray-level values. |
|  |  | **Large Area Low Gray Level Emphasis (LALGLE)** | $LALGLE= \frac{\sum_{i=1}^{N_{g}} \sum_{j=1}^{N_{s}} \frac{\mathbf{P}(i,j)j^{2}}{i^{2}}}{N_{z}}$ | LALGLE measures the proportion in the image of the joint distribution of larger size zones with lower gray-level values. |
|  |  | **Large Area High Gray Level Emphasis (LAHGLE)** | $LAHGLE= \frac{\sum_{i=1}^{N_{g}} \sum_{j=1}^{N_{s}} \mathbf{P}(i,j)i^{2}j^{2}}{N_{z}}$ | LAHGLE measures the proportion in the image of the joint distribution of larger size zones with higher gray-level values. |
|  | **Gray Level Size Zone Matrix** (**GLSZM**, 16 features)  ⚫$N_{g}$ be the number of discrete intensity values in the image  ⚫$N_{r}$ be the number of discrete run lengths in the image  ⚫$N_{p}$ be the number of voxels in the image  ⚫$N_{r}(\theta)$ be the number of runs in the image along angle θ, which is equal to $\sum_{i=1}^{N_{g}} \sum_{j=1}^{N_{r}} \mathbf{P}(i,j\vert\theta)$ and 1≤$N_{r}(\theta)$≤$N_{p}$  ⚫$\mathbf{P}(i,j\vert\theta)$ be the run length matrix for an arbitrary direction θ  ⚫$p(i,j\vert\theta)$ be the normalized run length matrix, defined as $p\left( i,j \vert\theta\right)=\frac{\mathbf{P}(i,j\vert\theta)}{N_{r}(\theta)}$ | **Short Run Emphasis (SRE)** | $SRE=\frac{\sum_{i=1}^{N_{g}} \sum_{j=1}^{N_{r}} \frac{\mathbf{P}(i,j\vert\theta)}{j^{2}}}{N_{r}(\theta)}$ | SRE is a measure of the distribution of short run lengths |
|  |  | **Long Run Emphasis (LRE)** | $SRE=\frac{\sum_{i=1}^{N_{g}} \sum_{j=1}^{N_{r}} \mathbf{P}(i,j\vert\theta)j^{2}}{N_{r}(\theta)}$ | LRE is a measure of the distribution of long run lengths |
|  |  | **Gray Level Non-Uniformity (GLN)** | $GLN=\frac{\sum_{i=1}^{N_{g}} {(\sum_{j=1}^{N_{r}} \mathbf{P}(i,j\vert\theta))}^{2}}{N_{r}(\theta)}$ | GLN measures the similarity of gray-level intensity values in the image |
|  |  | **Gray Level Non-Uniformity Normalized (GLNN)** | $GLNN=\frac{\sum_{i=1}^{N_{g}} {(\sum_{j=1}^{N_{r}} \mathbf{P}(i,j\vert\theta))}^{2}}{{N_{r}(\theta)}^{2}}$ | GLNN measures the similarity of gray-level intensity values in the image |
|  |  | **Run Length Non-Uniformity (RLN)** | $GLN=\frac{\sum_{i=1}^{N_{r}} {(\sum_{j=1}^{N_{g}} \mathbf{P}(i,j\vert\theta))}^{2}}{N_{r}(\theta)}$ | RLN measures the similarity of run lengths throughout the image |
|  |  | **Run Length Non-Uniformity Normalized (RLNN)** | $RLNN=\frac{\sum_{i=1}^{N_{r}} {(\sum_{j=1}^{N_{g}} \mathbf{P}(i,j\vert\theta))}^{2}}{{N_{r}(\theta)}^{2}}$ | RLNN measures the similarity of run lengths throughout the image |
|  |  | **Run Percentage (RP)** | $RP=\frac{N_{r}(\theta)}{N_{p}}$ | RP measures the coarseness of the texture by taking the ratio of number of runs and number of voxels in the ROI. |
|  |  | **Gray Level Variance (GLV)** | $GLV=\sum_{i=1}^{N_{g}} \sum_{j=1}^{N_{r}} p(i,j\vert\theta){(i-\mu)}^{2}$  $Here, \mu=\sum_{i=1}^{N_{g}} \sum_{j=1}^{N_{r}} p(i,j\vert\theta)i$ | GLV measures the variance in gray level intensity for the runs. |
|  |  | **Run Variance (RV)** | $RV=\sum_{i=1}^{N_{g}} \sum_{j=1}^{N_{r}} p(i,j\vert\theta){(j-\mu)}^{2}$  $Here, \mu=\sum_{i=1}^{N_{g}} \sum_{j=1}^{N_{r}} p(i,j\vert\theta)j$ | RV is a measure of the variance in runs for the run lengths. |
|  |  | **Run Entropy (RE)** | $RE=-\sum_{i=1}^{N_{g}} \sum_{j=1}^{N_{r}} p\left( i,j \vert\theta\right){log}_{2} (p\left( i,j \vert\theta\right)+\epsilon)$ | RE measures the uncertainty/randomness in the distribution of run lengths and gray levels. |
|  |  | **Low Gray Level Run Emphasis (LGLRE)** | $LGLRE=\frac{\sum_{i=1}^{N_{g}} \sum_{j=1}^{N_{r}} \frac{\mathbf{P}(i,j\vert\theta)}{i^{2}}}{N_{r}(\theta)}$ | LGLRE measures the distribution of low gray-level values. |
|  |  | **High Gray Level Run Emphasis (HGLRE)** | $HGLRE=\frac{\sum_{i=1}^{N_{g}} \sum_{j=1}^{N_{r}} \mathbf{P}(i,j\vert\theta)i^{2}}{N_{r}(\theta)}$ | HGLRE measures the distribution of the higher gray-level values. |
|  |  | **Short Run Low Gray Level Emphasis (SRLGLE)** | $SRLGLE=\frac{\sum_{i=1}^{N_{g}} \sum_{j=1}^{N_{r}} \frac{\mathbf{P}(i,j\vert\theta)}{i^{2}j^{2}}}{N_{r}(\theta)}$ | SRLGLE measures the joint distribution of shorter run lengths with lower gray-level values. |
|  |  | **Short Run High Gray Level Emphasis (SRHGLE)** | $SRHGLE=\frac{\sum_{i=1}^{N_{g}} \sum_{j=1}^{N_{r}} \frac{\mathbf{P}(i,j\vert\theta)i^{2}}{j^{2}}}{N_{r}(\theta)}$ | SRHGLE measures the joint distribution of shorter run lengths with higher gray-level values. |
|  |  | **Long Run Low Gray Level Emphasis (LRLGLE)** | $LRLGLRE=\frac{\sum_{i=1}^{N_{g}} \sum_{j=1}^{N_{r}} \frac{\mathbf{P}(i,j\vert\theta)j^{2}}{i^{2}}}{N_{r}(\theta)}$ | LRLGLRE measures the joint distribution of long run lengths with lower gray-level values. |
|  |  | **Long Run High Gray Level Emphasis (LRHGLE)** | $LRHGLRE=\frac{\sum_{i=1}^{N_{g}} \sum_{j=1}^{N_{r}} \mathbf{P}(i,j\vert\theta)i^{2}j^{2}}{N_{r}(\theta)}$ | LRHGLRE measures the joint distribution of long run lengths with higher gray-level values. |
|  | **Neighbouring Gray Tone Difference Matrix** (**NGTDM**, 5 features)  ⚫$n_{i}$ be the number of voxels in $X_{gl}$ with gray level i  ⚫$N_{v,p}$ be the total number of voxels in $X_{gl}$ and equal to $\sum n_{i}$ (i.e. the number of voxels with a valid region; at least 1 neighbor). $N_{v,p}$≤$N_{p}$, where $N_{p}$ is the total number of voxels in the ROI.  ⚫$p_{i}$ be the gray level probability and equal to ${n_{i}/N}_{p}$  ⚫$s_{i}=\left\{ \begin{aligned} \sum^{n_{i}} \left\vert i-\bar{A_{i}} \right\vert\mathrm{for} n_{i}\neq0 \\ 0 for n_{i}=0 \end{aligned} \right.$  be the sum of absolute differences for gray level ii  ⚫$N_{g}$ be the number of discrete gray levels  ⚫$N_{g,p}$ be the number of gray levels where $p_{i}$≠0 | **Coarseness** | $Coarseness=\frac{1}{\sum_{i=0}^{N_{g}} p_{i}s_{i}}$ | Coarseness is a measure of average difference between the center voxel and its neighbourhood and is an indication of the spatial rate of change. |
|  |  | **Contrast** | $Contrast=\left( \frac{1}{N_{g,p}(N_{g,p}-1)}\sum_{i=1}^{N_{g}} \sum_{j=1}^{N_{g}} p_{i}p_{j}{(i-j)}^{2} \right)\left( \frac{1}{N_{v,p}}\sum_{i=1}^{N_{g}} s_{i} \right),\mathrm{where} p_{i}\neq0,p_{j}\neq0$ | Contrast is a measure of the spatial intensity change, but is also dependent on the overall gray level dynamic range. |
|  |  | **Busyness** | $Busyness=\frac{\sum_{i=1}^{N_{g}} p_{i}s_{i}}{\sum_{i=1}^{N_{g}} \sum_{j=1}^{N_{g}} \left\vert{ip}_{i}{-jp}_{j} \right\vert},\mathrm{where} p_{i}\neq0,p_{j}\neq0$ |  |
|  |  | **Complexity** | $Complexity=\frac{1}{N_{v,p}}\sum_{i=1}^{N_{g}} \sum_{j=1}^{N_{g}} \left\vert i-j \right\vert\frac{p_{i}s_{i}+p_{j}s_{j}}{p_{i}+p_{j}},\mathrm{where} p_{i}\neq0,p_{j}\neq0$ |  |
|  |  | **Strength** | $Strength=\frac{\sum_{i=1}^{N_{g}} \sum_{j=1}^{N_{g}} \left( p_{i}{+p}_{j} \right)\left( i-j \right)^{2}}{\sum_{i=1}^{N_{g}} s_{i}},\mathrm{where} p_{i}\neq0,p_{j}\neq0$ | Strength is a measure of the primitives in an image. |
|  | **Gray Level Dependence Matrix** (**GLDM**, 14 features)  ⚫$N_{g}$ be the number of discrete intensity values in the image  ⚫$N_{d}$ be the number of discrete dependency sizes in the image  ⚫$N_{z}$ be the number of dependency zones in the image, which is equal to $\sum_{i=1}^{N_{g}} \sum_{j=1}^{N_{d}} \mathbf{P}(i,j)$  ⚫$\mathbf{P}(i,j)$ be the dependence matrix  ⚫$p(i,j)$ be the normalized dependence matrix, defined as $p\left( i,j \right)=\frac{\mathbf{P}(i,j)}{N_{z}}$ | **Small Dependence Emphasis (SDE)** | $SDE=\frac{\sum_{i=1}^{N_{g}} \sum_{j=1}^{N_{d}} \frac{\mathbf{P}(i,j)}{i^{2}}}{N_{z}}$ | A measure of the distribution of small dependencies. |
|  |  | **Large Dependence Emphasis (LDE)** | $LDE=\frac{\sum_{i=1}^{N_{g}} \sum_{j=1}^{N_{d}} \mathbf{P}(i,j)j^{2}}{N_{z}}$ | A measure of the distribution of large dependencies. |
|  |  | **Gray Level Non-Uniformity (GLN)** | $GLN=\frac{\sum_{i=1}^{N_{g}} \left( \sum_{j=1}^{N_{d}} \mathbf{P}(i,j) \right)^{2}}{N_{z}}$ | Measures the similarity of gray-level intensity values in the image. |
|  |  | **Dependence Non-Uniformity (DN)** | $DN=\frac{\sum_{j=1}^{N_{d}} \left( \sum_{i=1}^{N_{g}} \mathbf{P}(i,j) \right)^{2}}{N_{z}}$ | Measures the similarity of dependence throughout the image. |
|  |  | **Dependence Non-Uniformity Normalized (DNN)** | $DNN=\frac{\sum_{j=1}^{N_{d}} \left( \sum_{i=1}^{N_{g}} \mathbf{P}(i,j) \right)^{2}}{N_{z}^{2}}$ | Measures the similarity of dependence throughout the image. |
|  |  | **Gray Level Variance (GLV)** | $GLV=\sum_{i=1}^{N_{g}} \sum_{j=1}^{N_{d}} p\left( i,j \right)\left( i-\mu\right)^{2},$  $\mathrm{where}\mu=\sum_{i=1}^{N_{g}} \sum_{j=1}^{N_{d}} ip(i,j)$ | Measures the variance in grey level in the image. |
|  |  | **Dependence Variance (DV)** | $DV=\sum_{i=1}^{N_{g}} \sum_{j=1}^{N_{d}} p\left( i,j \right)\left( j-\mu\right)^{2},$  $\mathrm{where}\mu=\sum_{i=1}^{N_{g}} \sum_{j=1}^{N_{d}} jp(i,j)$ | Measures the variance in dependence size in the image. |
|  |  | **Dependence Entropy (DE)** | $DependenceEntropy=-\sum_{i=1}^{N_{g}} \sum_{j=1}^{N_{d}} p(i,j){log}_{2}(p\left( i,j \right)+\epsilon)$ |  |
|  |  | **Low Gray Level Emphasis (LGLE)** | $LGLE=\frac{\sum_{i=1}^{N_{g}} \sum_{j=1}^{N_{d}} \frac{\mathbf{P}(i,j)}{i^{2}}}{N_{z}}$ | Measures the distribution of low gray-level values |
|  |  | **High Gray Level Emphasis (HGLE)** | $HGLE=\frac{\sum_{i=1}^{N_{g}} \sum_{j=1}^{N_{d}} \mathbf{P}(i,j)i^{2}}{N_{z}}$ | Measures the distribution of the higher gray-level values. |
|  |  | **Small Dependence Low Gray Level Emphasis (SDLGLE)** | $SDLGLE=\frac{\sum_{i=1}^{N_{g}} \sum_{j=1}^{N_{d}} \frac{\mathbf{P}(i,j)}{i^{2}j^{2}}}{N_{z}}$ | Measures the joint distribution of small dependence with lower gray-level values. |
|  |  | **Small Dependence High Gray Level Emphasis (SDHGLE)** |  | Measures the joint distribution of small dependence with higher gray-level values. |
|  |  | **Large Dependence Low Gray Level Emphasis (LDLGLE)** | $LDLGLE=\frac{\sum_{i=1}^{N_{g}} \sum_{j=1}^{N_{d}} \frac{\mathbf{P}(i,j)j^{2}}{i^{2}}}{N_{z}}$ | Measures the joint distribution of large dependence with lower gray-level values. |
|  |  | **Large Dependence High Gray Level Emphasis (LDHGLE)** | $LDHGLE=\frac{\sum_{i=1}^{N_{g}} \sum_{j=1}^{N_{d}} \mathbf{P}(i,j){i^{2}j}^{2}}{N_{z}}$ | Measures the joint distribution of large dependence with higher gray-level values. |

1. van Griethuysen, J.J.M., A. Fedorov, C. Parmar, et al., Computational Radiomics System to Decode the Radiographic Phenotype. Cancer Res, 2017; 77(21): e104-e107. dio: 10.1158/0008-5472.Can-17-0339.
